# Repetitive transcranial magnetic stimulation alleviates neuropathic pain via microglial polarization by modulating the METTL3/NMDAR2B/NLRP3 pathway

**DOI:** 10.3389/fimmu.2025.1666920

**Published:** 2026-01-26

**Authors:** Jiayi Zhu, Lei Li, Rongnan Shi, Fei Xing, Yue Yang, Zhangyu Xu, Qin Wang, Qi Deng, Dan Li, Maomao Huang, Jianxiong Wang

**Affiliations:** 1 Rehabilitation Medicine Department, The Affiliated Hospital of Southwest Medical University, Luzhou, Sichuan, China; 2 Rehabilitation Medicine and Engineering Key Laboratory of Luzhou, Luzhou, Sichuan, China

**Keywords:** neuropathic pain, repetitive transcranial magnetic stimulation, METTL3, NMDAR2B, NLRP3, microglial polarization

## Abstract

**Introduction:**

Neuropathic pain (NeuP) remains a major clinical condition, and the existing interventions often fail to provide sufficiently satisfactory symptom control. Repetitive transcranial magnetic stimulation (rTMS) has gained attention as a potential non-invasive therapy for NeuP. However, the precise mechanisms underlying the effects of rTMS remain elusive.

**Objective:**

This study explores the potential neurophysiological mechanisms of rTMS in NeuP, focusing on its modulation of the methyltransferase-like 3 (METTL3)/*N*-methyl-d-aspartate receptor subtype 2B (NMDAR2B)/NOD-, LRR- and pyrin domain-containing protein 3 (NLRP3) axis and microglial polarization.

**Methods:**

CCI rats began to receive rTMS treatment once daily 7 days after the operation, and the treatment continued for 4 weeks. Pain and depression-like behaviors were evaluated by measuring the paw-withdrawal mechanical threshold (PWMT), thermal pain-induced paw-withdrawal latency (PWL), sciatic nerve function index (SFI), forced swimming test (FST) results, and new object preference index (NPI). The expression levels of relevant indicators were detected by immunofluorescence and western blot analyses, quantitative reverse transcription-polymerase chain reaction (qRT-PCR), and enzyme-linked immunosorbent assay (ELISA). BV2 microglia were cultured in Dulbecco’s modified Eagle medium. After adding agonists and inhibitors of METTL3 and NMDAR2B, the microglia were treated with lipopolysaccharide (LPS; 100 µg/mL) for 12 h. The cells are divided into seven groups: Control (Con), LPS, LPS + magnetic stimulation (MS), LPS + METTL3 inhibition (sh-METTL3), LPS + METTL3 overexpression + magnetic stimulation (LPS+METTL3-OE+MS), LPS + NMDAR2B inhibition (sh-NMDAR2B), and LPS + NMDAR2B overexpression + magnetic stimulation (LPS+NMDAR2B-OE+MS). The expression levels of cell polarization markers, inflammatory factors, and related proteins were detected by methods such as immunofluorescence and western blot analyses, qRT-PCR, and ELISA.

**Results:**

rTMS improved pain thresholds (PWMT, PWL, and SFI) and depressive-like behaviors, reduced immobility in the FST, and increased the NPI. It inhibited the levels of the pro-inflammatory markers interleukin (IL)-6, tumor necrosis factor (TNF)-α, NLRP3,TMEM119 and iNOS in the dorsolateral prefrontal cortex (DLPFC), while increasing the expression of IL-10 and Arg1. Moreover, rTMS decreased the expression levels of the M1-type marker CD86 of microglia and increased those of the M2-type marker CD206 and simultaneously decreased the expression of microglia activation marker Iba-1. rTMS simultaneously downregulated METTL3, N6-methyladenosine (m6A), NMDAR2B, and YTH domain-containing family 1 (YTHDF1). In the *in vitro* experiments, LPS-induced BV2 cells showed increased expression of CD86 increased (p < 0.01) as well as NLRP3, IL-6, TNF-α, and METTL3/m6A/YTHDF1/NMDAR2B (p < 0.01), and decreased expression of CD206 and IL-10. Magnetic stimulation reversed these effects, promoted the reduction of microglial marker Iba-1, increased M2 polarization and alleviated inflammation (p < 0.01). Inhibition of METTL3 or NMDAR2B alleviated LPS-induced inflammation. However, activation of METTL3 or NMDAR2B counteracted the effects of magnetic stimulation in improving inflammation (p < 0.01). In addition, suppressing or overexpressing METTL3, YTHDF1, and NMDAR2B correspondingly decreased or increased these effects, but modulation of NMDAR2B did not change the expression of METTL3/YTHDF1.

**Conclusion:**

rTMS can affect the polarization state of microglia and neuroinflammation by regulating the METTL3/NMDAR2B/NLRP3 signaling pathway, thereby improving NeuP.

## 1 Introduction

Neuropathic pain (NeuP) is a type of chronic pain that results directly from damage to or disease of the somatosensory nervous system (1, 2). NeuP accounts for approximately 7%–10% of all chronic pain cases (3, 4). The existing pharmacological treatments for NeuP include calcium channel blockers, tricyclic antidepressants, and opioids; however, approximately 40%–60% of patients still do not experience effective pain relief (5, 6). Moreover, chronic NeuP is frequently accompanied by comorbid mood disorders such as anxiety and depression as well as cognitive dysfunction (7–10), all of which severely impair patients’ quality of life and impose substantial socioeconomic burdens.

Repetitive transcranial magnetic stimulation (rTMS) is a non-invasive neuromodulation technique that has been widely applied to treat conditions such as paralysis, pain, spasticity, and cognitive impairment, which often arise secondary to stroke, spinal cord injury, traumatic brain injury, and similar conditions (11–15). Previous studies have demonstrated that rTMS targeting the primary motor cortex or the prefrontal cortex (PFC) can effectively alleviate various forms of chronic pain, including NeuP (16, 17). Microglia in the activated state can be classified into two types: the pro-inflammatory M1 type and the anti-inflammatory M2 type. An abnormal increase in M1-type microglia is a key cause of NeuP (18). Recent evidence suggests that shifting microglial polarization from the M1 to the M2 type represents one of promising therapeutic strategies for NeuP (19–21). Our previous study indicated that rTMS may alleviate NeuP by modulating microglial M1/M2 polarization and reducing neuroinflammation (22). Nevertheless, the precise molecular mechanisms underlying these effects require further investigation.

Epigenetic mechanisms, which regulate gene expression without altering DNA sequences, have been recognized as one of the key contributors to the pathogenesis of NeuP (23). N6-methyladenosine (m6A) is the most prevalent post-transcriptional modification in eukaryotic mRNA. The m6A modification is dynamically regulated by a complex system involving methyltransferases (such as methyltransferase-like 3 [METTL3] and methyltransferase-like 14 [METTL14]), demethylases (including fat mass- and obesity-associated protein [FTO] and AlkB homolog H5 [ALKBH5]), and recognition proteins (such as the proteins of the YTH domain-containing family [YTHDF]). Together, these regulators influence various aspects of mRNA metabolism, including splicing, transport, stability, and translation efficiency (24, 25). Recent studies have suggested that m6A methylation may play a crucial role in the development and maintenance of NeuP (26).

METTL3 is a critical m6A methyltransferase that plays an important role in NeuP by influencing neuroinflammatory and pain signaling pathways (27). Chen et al. reported elevated METTL3 expression in chronic constriction injury (CCI) models and improvement of pain-related behaviors after inhibition of METTL3 in CCI rats (28). A previous study found that METTL3 can regulate microglial M1/M2 polarization in rat models (29). The results of that study suggested that METTL3 may be a potential therapeutic target for NeuP. Xia et al. also found that S-adenosylhomocysteine (SAH; an inhibitor of the METTL3-METTL14 complex) reduces the expression of *N*-methyl-d-aspartate receptor subtype 2B (NMDAR2B) and alleviates pain in uterine cervical dilation model rats (30). NMDAR2B is a key subunit of excitatory glutamate receptors, which may promote microglial M1 polarization under some pathological conditions such as NeuP (31, 32) and play a crucial role in the transition phase of NeuP (33). Moreover, some studies have suggested that NMDAR2B may play a role in modulating the NOD-, LRR- and pyrin domain-containing protein 3 (NLRP3) inflammasome (34, 35), which is also involved in NeuP by triggering the maturation and release of pro-inflammatory cytokines such as interleukin (IL)-1β and IL-18 (36). Simultaneously, the NLRP3 inflammasome was found to be associated with microglia M1/M2 polarization in spinal cord injury models (37). These studies indicate that the METTL3/NMDAR2B/NLRP3 pathway may be involved in microglial polarization-mediated neuroinflammation.

A previous study showed that deep brain stimulation can alleviate post-traumatic stress disorder behavior by reducing the expression of METTL3 (38), and our earlier research demonstrated that rTMS reduces NMDAR2B expression to ameliorate NeuP and related behaviors (8). rTMS has been also shown to inhibit NLRP3 activation in several disease models (39). On the basis of these findings, we aimed to investigate the role of the METTL3/NMDAR2B/NLRP3 signaling pathway in the therapeutic effects of rTMS on NeuP.

This study initially examined the effects of rTMS on the METTL3/NMDAR2B/NLRP3 axis using a rat model of CCI-induced NeuP. Subsequently, the biological effects of magnetic stimulation observed *in vivo* were confirmed in an *in vitro* neuroinflammation model in which M1 polarization of BV microglia was induced by lipopolysaccharide (LPS). Finally, by inhibiting or activating METTL3/NMDAR2B, we aimed to clarify its role in the regulation of microglial polarization and neuroinflammation by magnetic stimulation. We hope our findings may offer some additional insights into the mechanisms of rTMS on NeuP.

## 2 Materials and methods

### 2.1 Experimental animals

Adult male Sprague–Dawley (SD) rats, aged 8 weeks and weighing between 200–240 g, were obtained from the Laboratory Animal Center of Southwest Medical University. A total of 15 rats were used in this study, with the sample size determined on the basis of the number of experimental groups and the required animals per group. Animals were randomly allocated into three groups: Control (n = 5), CCI+Sham (n = 5), and CCI+rTMS (n = 5). Related behavioral assessments were conducted at designated time points. After completion of behavioral testing, the rats were euthanized for the analysis of relevant molecular indicators. The Animal Ethics Committee of Southwest Medical University approved all experimental procedures. Behavioral testing and image quantification were performed by two investigators blinded to group allocation; random numeric codes were revealed only after statistical analysis.

### 2.2 Animal model

**Figure 1:**
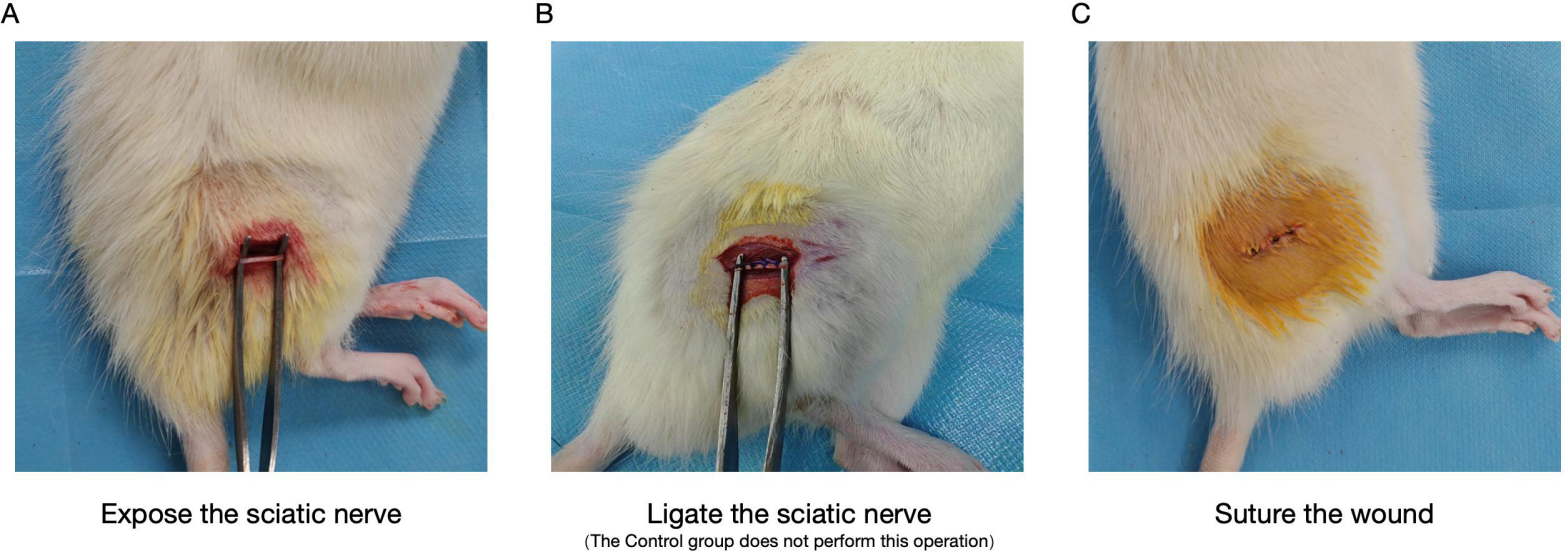
Schematic diagram of animal modeling.

**Figure 2:**
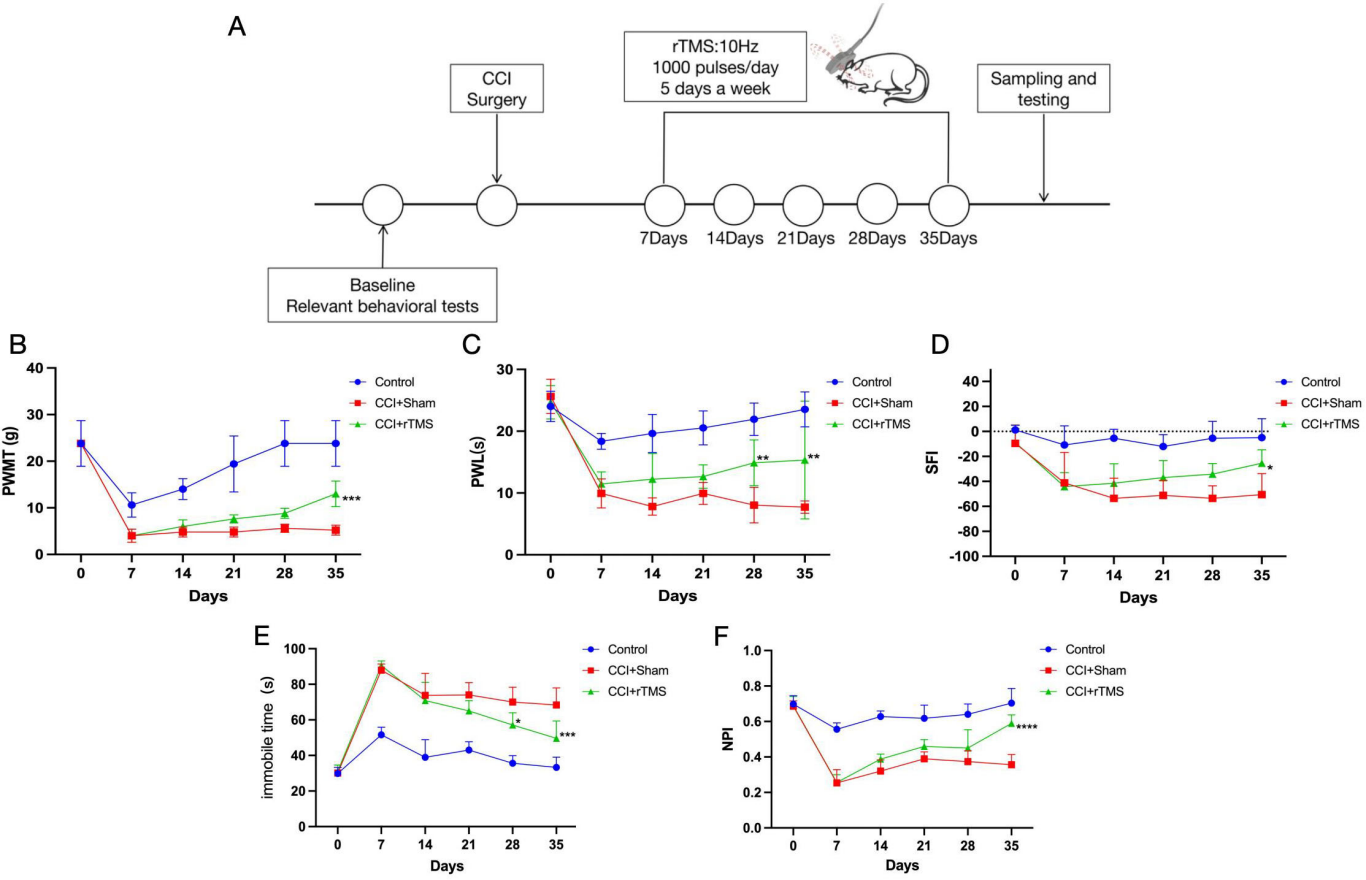
The influence of rTMS treatment on pain and related depressive behaviors. **(A)** The timing of the experimental procedures. **(B)** The changes in the PWMT of rats in each group from 1 to 5 weeks after operation. **(C)** The changes in the PWL of rats in each group from 1 to 5 weeks after operation. **(D)** Change trends in the rat SFI. **(E)** Time variations in the swimming immobility time. **(F)** Changes in the NPI. The number of samples in each group was 5; the results was analyzed with one-way ANOVA followed by Tukey’s *post-hoc* test, *CCI+Sham group vs. CCI+rTMS group, p < 0.05, **P < 0.01, ***P < 0.001 and ****P < 0.0001.

The CCI and Control models were established according to previously reported protocols. Briefly, rats were anesthetized with an intraperitoneal injection of 1.0% pentobarbital sodium at a dose of 40 mg/kg. After anesthesia, the rat was placed in a prone position on the operating table. The surgical area was shaved and disinfected with iodine and 75% ethanol. A skin incision was made on the left mid-thigh, and the underlying subcutaneous tissue was carefully dissected. A parallel incision was made approximately 3–4 mm below the right femur, and the muscle was gently separated to expose the sciatic nerve trunk. At the proximal segment of the nerve, four loose ligations were placed using 4–0 silk, spaced 1 mm apart. These ligations were tight enough to constrict the nerve partially and occasionally elicited a brief muscle twitch. In the Sham group, the sciatic nerve was exposed but not ligated. Finally, the incision was closed in layers using 4–0 non-absorbable sutures. All animals were monitored daily following surgery. A schematic illustration of the model is shown in **Figure 1**, and the timeline of behavioral testing is presented in **Figure 2A**. Twenty‐four hours after the last stimulation session and final behavioral test, rats were euthanized by intraperitoneal injection of sodium pentobarbital(60mg/kg).

### 2.3 Cell culture and treatment

BV2 microglial cells (JK-CS0548; ATCC) were cultured in Dulbecco’s modified Eagle medium (DMEM; 6124560, Gibco) supplemented with 5% fetal bovine serum (FBS; 11011-8611; Every Green) and 0.5% penicillin-streptomycin solution (100×; C0222; Beyotime). Cells were incubated at 37 °C in a humidified atmosphere containing 5% CO_2_. After seeding, BV2 cells were cultured in complete DMEM for 24 h. Subsequently, specific pharmacological activation and inhibition targeting METTL3 (METTL3 activator-1; HY-W037893; MedChemExpress; STM2457; T9060; Targetmol) and NMDAR2B (d-Serine; HY-100808; MedChemExpress; D-AP5; HY-100714A; MedChemExpress) were added to the culture medium, and their effects were verified. BV2 cells were pretreated with STM2457 (10 µM, 2 h) or METTL3-activator-1 (5 µM, 2 h) for METTL3 modulation, and D-AP5 (50 µM, 1 h) or D-Serine (100 µM, 1 h) for NMDAR2B modulation, followed by LPS (100 µg/mL, 12 h) before magnetic stimulation and downstream analyses. The cells were then randomly allocated into the following experimental groups: Control (Con), LPS, LPS combined with magnetic stimulation, LPS with METTL3 knockdown (sh-METTL3), LPS with METTL3 overexpression and magnetic stimulation (METTL3-OE+MS), LPS with NMDAR2B inhibition (sh-NMDAR2B), and LPS with NMDAR2B overexpression and magnetic stimulation (NMDAR2B-OE+MS). After the final magnetic-pulse treatment, BV2 cells were cultured for an additional 6 h, then fixed and harvested for further analyses.

### 2.4 Repetitive transcranial magnetic stimulation

Seven days after the operation, rTMS was administered to rats in the rTMS group for four consecutive weeks (5 days/week) using a circular coil (6 cm diameter, 3 T peak intensity; YRD CCY-I, Wuhan, China). Each rat was gently restrained on a custom platform with a 3-day acclimation period to minimize movement (40). PFC targeting was validated across animals through: 1) stereotaxic coordinates (AP + 3.2 mm, ML + 0.7 mm, DV -1.5 mm relative to bregma) verified against anatomical landmarks (41); 2) resting motor threshold (RMT) determination via forepaw MEPs to standardize intensity (100% RMT = 40% max output) Stimulation parameters included 10 Hz frequency, 20 trains (20 s duration, 10 s interval), and 1,000 pulses/session. Control animals received identical restraint and auditory cues without magnetic stimulation (30 cm from coil). This comprehensive approach ensured accurate, reproducible targeting while addressing inter-animal variability in skull thickness and brain size (42).

### 2.5 *In vitro* magnetic stimulation treatment

After establishment of the *in vitro* model, BV2 cells were seeded into T75 culture flasks, with each flask receiving an appropriate volume of complete growth medium. Magnetic stimulation was applied using a Magstim device (YRD CCY-I; Wuhan, China). The stimulation protocol consisted of 960 trains delivered over 48 h, with sessions repeated every 24 h. Each train was administered at a frequency of 10 Hz, lasted for 2 s, and was followed by a 5.5-s interval. The stimulation parameters were set at 500 µV/div and 5 ms/div using the device’s default settings. Control plates were placed in an identical incubator equipped with an inactive coil to compensate for any temperature or vibration effects (43, 44).

### 2.6 Paw-withdrawal mechanical threshold

To minimize stress-related interference, the rats were habituated to the testing environment for 30 min daily, starting three days before surgery. This acclimation period was also repeated for 30 min immediately before behavioral assessments conducted at baseline (one day before surgery) and on postoperative days 7, 14, 21, 28, and 35. For paw-withdrawal mechanical threshold (PWMT) testing, each rat was placed in a transparent plastic chamber with a wire mesh floor and allowed to settle for approximately 15 min until it become calm. Mechanical stimulation was then applied perpendicular to the plantar surface of the right hind paw, specifically between the second and third metatarsals, using a series of Von Frey filaments (Aesthesio; Shenzhen RWD Life Science Co., Ltd.) of increasing stiffness. Each stimulus was applied for approximately 5 s, with several seconds of rest between trials. A brisk paw withdrawal or licking response was recorded as a positive reaction. When the rats showed ambiguous responses, the stimulus was repeated. The mechanical threshold was defined as the minimum force (in grams) that elicited a withdrawal response in at least three out of five consecutive applications.

### 2.7 Paw-withdrawal latency

To assess thermal pain sensitivity, we employed the hot plate test as described previously. Rats were individually placed on a metal surface maintained at 55 °C ± 0.5 °C, and the time until they either licked their paws or attempted to escape by jumping was recorded as the paw-withdrawal latency (PWL). To avoid tissue injury, animals that did not respond within 30 s were immediately removed from the plate and assigned a maximum latency of 30 s. Behavioral testing was conducted at baseline (one day prior surgery) and on postoperative days 7, 14, 21, 28, and 35.

### 2.8 Forced swimming test

The forced swimming test was also performed at the same time points. Each rat was placed in a transparent cylindrical tank (20 cm in diameter, 45 cm in height) filled with water to a depth of 30 cm and maintained at a temperature of 25 °C–27 °C. Before the formal test, the animals underwent a 15-min habituation swim session. Twenty-four hours later, immobility behavior was recorded during a 6-min test period. Immobility was defined as the absence of active struggling, with the animal remaining motionless except for minimal movements required to keep its head above the water surface.

### 2.9 Novel object preference index

During the habituation phase, rats were individually placed in an empty open-field chamber for 15 min per day over three consecutive days to acclimate them to the testing environment. After a 24-h rest period following the last habituation session, the training phase began. Two identical objects were placed in the chamber, and the rat was allowed to explore each object for 10 min. One hour later, one of the objects was randomly replaced with a novel item, and the rat was given an additional 5 min to explore the novel object. Between trials, the chamber and objects were cleaned with 70% ethanol to eliminate olfactory cues. Exploration was defined as sniffing or touching the object with the forepaws; climbing or sitting on the object was not counted. The novel object preference index (NPI) was calculated as the time spent exploring the novel object divided by the total time spent exploring both objects.

### 2.10 Sciatic nerve function index

The sciatic nerve function index (SFI) was assessed at baseline (1 day before surgery) and on postoperative days 7, 14, 21, 28, and 35. A walking track was constructed using plastic panels on both sides and a dark box at the end. The floor was lined with clean white paper, and non-toxic ink was applied to the hind paws of the rats. Each rat was placed at the start of the track and allowed to walk freely into the dark box, leaving footprints on the paper as it moved. Three clear footprints were selected from each rat, and the following parameters were measured on both the normal (N) and operated (E) sides (1): distance from the heel to third toe (NPL and EPL) (2), distance from the first to fifth toe (NTS and ETS), and (3) distance from the second to fourth toe (NIT and EIT). The SFI was calculated using the formula:


SFI=−38.3(EPL−NPL/NPL)+109.5(ETS−NTS/NTS)+13.3(EIT−NIT/NIT)−8.8

In healthy rats, the SFI value was approximately 0, whereas complete sciatic nerve transection results in an SFI close to −100.

### 2.11 Immunofluorescence analyses

Tissue samples were fixed and subsequently sectioned using a cryostat. For antigen retrieval, sections were incubated in either Tris-EDTA buffer (pH 9.0) or citrate buffer (pH 6.0; G1201; Servicebio), depending on the antibody requirements, and processed using a microwave-based retrieval system (BL617A; Biosharp). After cooling to room temperature, nonspecific binding was blocked by applying 5% normal goat serum (diluted 1:19 in PBS; SL038; Solarbio) for 30 min, followed by gentle washing. Primary antibodies against CD86 (1:100; bs-1035r; Bioss), CD206 (1:100; #24595; CST) and Iba-1(1:100, ab283319, Abcam, Cambridge, UK)were applied, and the sections were incubated overnight at 4 °C. After washing, the sections were incubated with appropriate secondary antibodies: Cy™3-conjugated goat anti-rabbit IgG (GB113497; Jackson) and fluorescein isothiocyanate (FITC)-conjugated goat anti-rabbit IgG (GB22303; Servicebio) for 50 min at room temperature. After additional washes with PBS, the nuclei were counterstained with DAPI (111-165-003; Biosharp) for 15 min. Finally, the sections were mounted using an anti-fade fluorescence mounting medium.

### 2.12 Quantitative image analysis

Background subtracted: ImageJ, rolling-ball 25 px. Positivity cut-off = mean fluorescence of 20 negative cells + 3 SD, applied uniformly across all images. *In vivo*: three 20× ROIs per animal, dorsolateral pre-frontal cortex (≥ 150 Iba-1^+^ cells/ROI). *In vitro*: ten random 20× fields per coverslip, three independent BV2 cultures (≥ 200 Iba-1^+^ cells/field).

### 2.13 Western blot analyses

Total protein was extracted from cells or tissue samples using radioimmunoprecipitation assay (RIPA) lysis buffer (G2002; Servicebio) supplemented with a protease and phosphatase inhibitor cocktail. Protein concentrations were determined using a bicinchoninic acid (BCA) assay kit (G2002; Servicebio). Equal amounts of protein were loaded onto sodium dodecyl sulfate-polyacrylamide gel electrophoresis (SDS-PAGE) gels and transferred onto polyvinylidende difluoride (PVDF) membranes (IPVH00010; Millipore). Membranes were blocked with 5% non-fat milk for 1 h at room temperature, followed by overnight incubation at 4 °C with primary antibodies against METTL3 (1:1000; A19079; Abclonal), NMDAR2B (1:1000; AF6426; Affinity), YTHDF1 (1:1000; 17479-1-AP; Proteintech), and β-actin (1:100,000; 81115-1-RR; Proteintech). After washing, membranes were incubated with horseradish peroxidase (HRP)-conjugated secondary antibodies for 1 h. Protein bands were visualized using an ECL detection kit (BL520A; Biosharp) and analyzed using ImageJ software.

### 2.14 Quantitative real-time polymerase chain reaction

For reverse transcription-polymerase chain reaction (RT-PCR) analysis, 1 mL of TRIzol reagent was added to 50–100 mg of tissue sample, and the mixture was incubated at 15–30 °C for 5 min to ensure complete dissociation of nucleoprotein complexes. Total RNA was extracted using the RNAsimple Kit (DP419; Tiangen), and complementary DNA (cDNA) was synthesized using the All-in-One 5×RT Master Mix (G592; ABM). Quantitative PCR was performed using Blastaq™ 2× qPCR MasterMix (G891; ABM). The primer designs are listed in **Table 1**.

**Table 1:** Forward and reverse primer sequences for target genes.

Gene	Direction	Primer sequence
*TMEM119*	Forward	CACAAGGCCACAGCCTACTA
	Reverse	ACCGGAGGTTCTGAGTAGCA
*iNOS*	Forward	GCATTCAGATCCCGAAACGC
	Reverse	GCCCTCGAAGGTGAGTTGAA
*Arg1*	Forward	CCAAGCCAAAGCCCATAGAGAT
	Reverse	CAGGCCAGCTTTCCTTAATGC
*METTL3*	Forward	ACACGTGGAGCTCTATCCAG
	Reverse	GCACTGGGCTATCACTACGG
*NMDAR2B*	Forward	CCTGAAAGACAAGGGCCGAT
	Reverse	GGTCAGGGTAGAGCGACTTG
*YTHDF1*	Forward	GACCCTCCCATCCCGTATCT
	Reverse	GGGAGCTTGGTGGGTAAGTG
*β-actin*	Forward	CCACTGCCGCATCCTCTT
	Reverse	GCATCGGAACCGCTCATT

### 2.15 Enzyme-linked immunosorbent assays

At the end of the experimental period, the rats were euthanized, and their brain tissues were collected for biochemical analysis. Tissue samples were weighed and rinsed with PBS to remove residual blood. PBS was added at a 1:9 (w/v) ratio, and tissues were homogenized using a mechanical grinder. The homogenate was centrifuged at 10,000 × *g* for 5–10 min, and the supernatant was collected for further analysis. For enzyme-linked immunosorbent assays (ELISAs), standards and samples were added to a 96-well plate pre-coated with capture antibodies (MM-71155R2 Rat m6A ELISA Kit, Rat IL-6 ELISA Kit, Rat TNF-α ELISA Kit, and Rat IL-10 ELISA Kit; all from Bioswamp). After incubation, HRP-conjugated secondary antibodies were added, followed by thorough washing to remove unbound components. The reaction was terminated by adding 50 μL of stop solution, and the absorbance at 450 nm was measured within 10 min using a microplate reader (Detielab HBS-1096A).

### 2.16 Statistical analysis

All data are presented as mean ± standard deviation (SD). Statistical analysis was performed using SPSS 24.0 (IBM, Armonk, NY, USA). Comparisons among multiple groups were analyzed using one-way analysis of variance (ANOVA) followed by the least significant difference (LSD) *post-hoc* test for normally distributed data or the Kruskal–Wallis test for non-normally distributed data. A p-value < 0.05 or <0.01 was considered statistically significant.

## 3 Results

### 3.1 rTMS improves pain and depression-related behaviors and promotes sciatic nerve recovery

Before the operation, the three groups (Control, CCI+Sham, and CCI+rTMS) showed no significant differences in PWMT, PWL, swimming immobility time, NPI, and SFI (**Figures 2B–F**). At 1 week post-modeling, in comparison with the Control group, the CCI+Sham and CCI+rTMS groups exhibited significantly reduced PWMT, PWL, and SFI (p < 0.01), indicating successful establishment of the NeuP model. Concurrently, the CCI and rTMS groups exhibited increased swimming immobility time and decreased NPI values (p < 0.01), indicating depressive-like behaviors in the rats.

At 2 to 5 weeks after the operation, the CCI+Sham group demonstrated significantly lower PWMT, PWL, and SFI than the Control group (p < 0.01). In the CCI+rTMS group, PWMT, PWL, and SFI began to increase after 1 week of intervention. By the third week, the PWL in the CCI+rTMS group was significantly higher than that in the CCI+Sham group (p < 0.01), and by the fourth week, the PWMT and SFI in the CCI+rTMS group were markedly greater than those in the CCI+Sham group (p < 0.05). Additionally, in comparison with the corresponding values in the CCI+Sham group, the CCI+rTMS group exhibited a shorter swimming immobility time after 3 weeks (p < 0.01) and a higher NPI after 4 weeks.

### 3.2 Magnetic stimulation shifts microglia from the M1 to M2 type and regulates neuroinflammation

**Figure 3:**
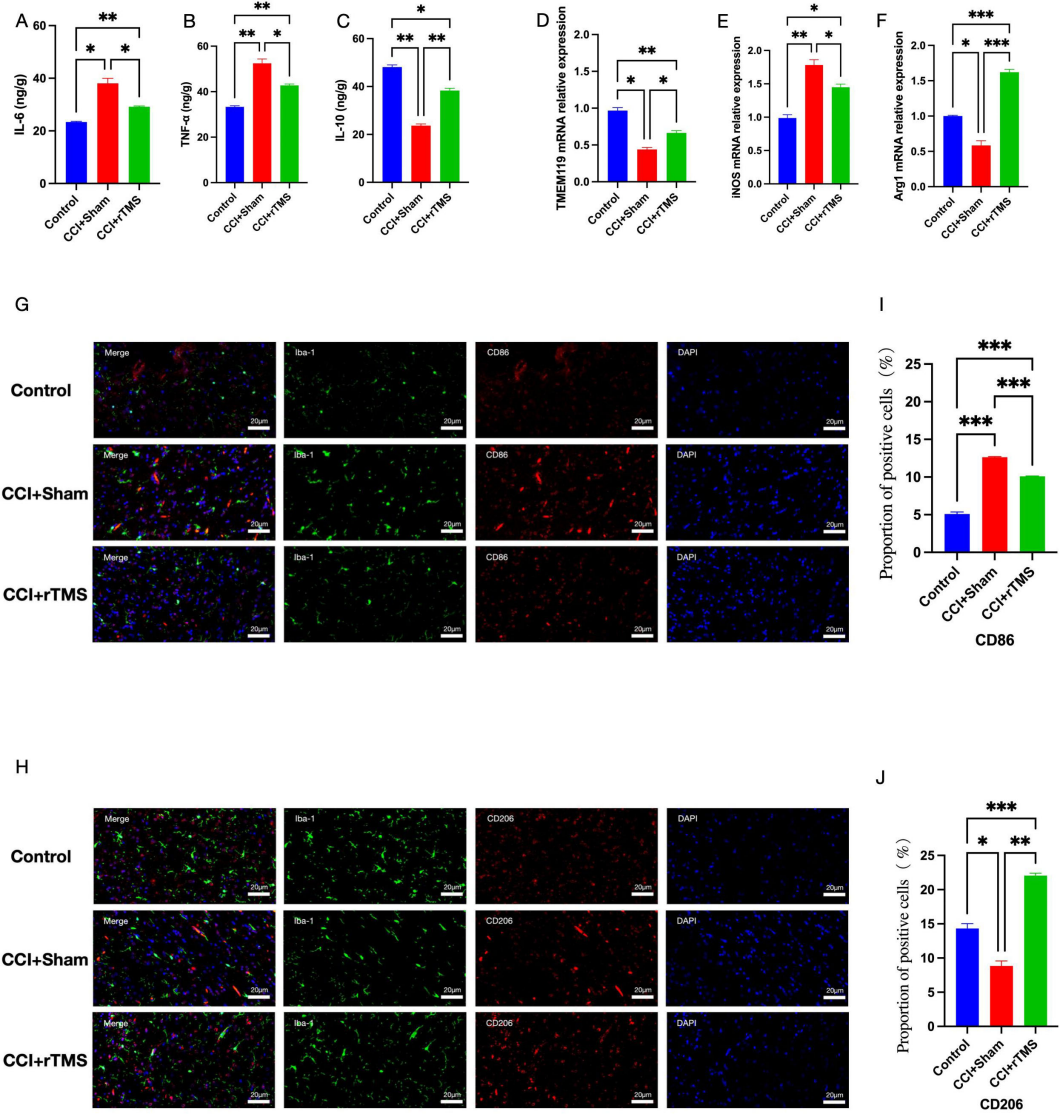
Effects of rTMS on neuroinflammation and the M1/M2 polarization state of microglia *in vivo*. **(A–C)** Elisa was used to examine the expression levels of IL-6, TNF-α and IL-10 expression level.**(D–F)** Real-time qRT-PCR analysis of the relative mRNA expression of *TMEM119*, *iNOS*, and *Arg1* in the DLPFC. **(G, H)** Immunofluorescence staining of Iba-1, M1-type (CD86) and M2-type (CD206) markers in the left PFC region. **(I, J)** Proportion of positive cells for target proteins. The number of samples in each group is 3. The results was analyzed with one-way ANOVA followed by Tukey’s *post-hoc* test, *p < 0.05, **P < 0.01 and ***P < 0.001.

*In vivo* experiments revealed that at 5 weeks post-modeling, the CCI+Sham group showed higher levels of IL-6 and TNF-α (p < 0.01) and lower IL-10 levels (p < 0.01) in the dorsolateral PFC (DLPFC) in comparison with the corresponding levels in the Control group (**Figures 3A–C**). Compared with the Sham group, the levels of *iNOS* in the CCI+Sham group increased, while the level of *TMEM119* and *Arg1* decreased(**Figures 3D–F**). Moreover, the CCI+Sham group showed increased proportion of the M1 phenotype marker CD86 (p < 0.01) and decreased proportion of the M2 phenotype marker CD206 (p < 0.01) in the DLPFC (**Figures 3G–J**). Thus, in the NeuP model, microglia predominantly polarized toward the M1 phenotype, thereby inducing neuroinflammation. After 4 weeks of the rTMS intervention, the CCI+rTMS group showed significantly lower levels of IL-6 and TNF-α (p < 0.01) and higher levels of IL-10 (p < 0.01) than the CCI+Sham group. Moreover, Compared with the CCI+Sham group, the levels of *iNOS* in the CCI+rTMS group decreased, while the level of *TMEM119* and *Arg1* increased, and the CCI+rTMS group exhibited reduced CD86 proportion (p < 0.01) and elevated CD206 levels (p < 0.01). Thus, rTMS may inhibit microglial polarization toward the M1 phenotype and promote its polarization toward the M2 phenotype, thereby returning to the “homeostasis” and regulating neuroinflammation.

**Figure 4:**
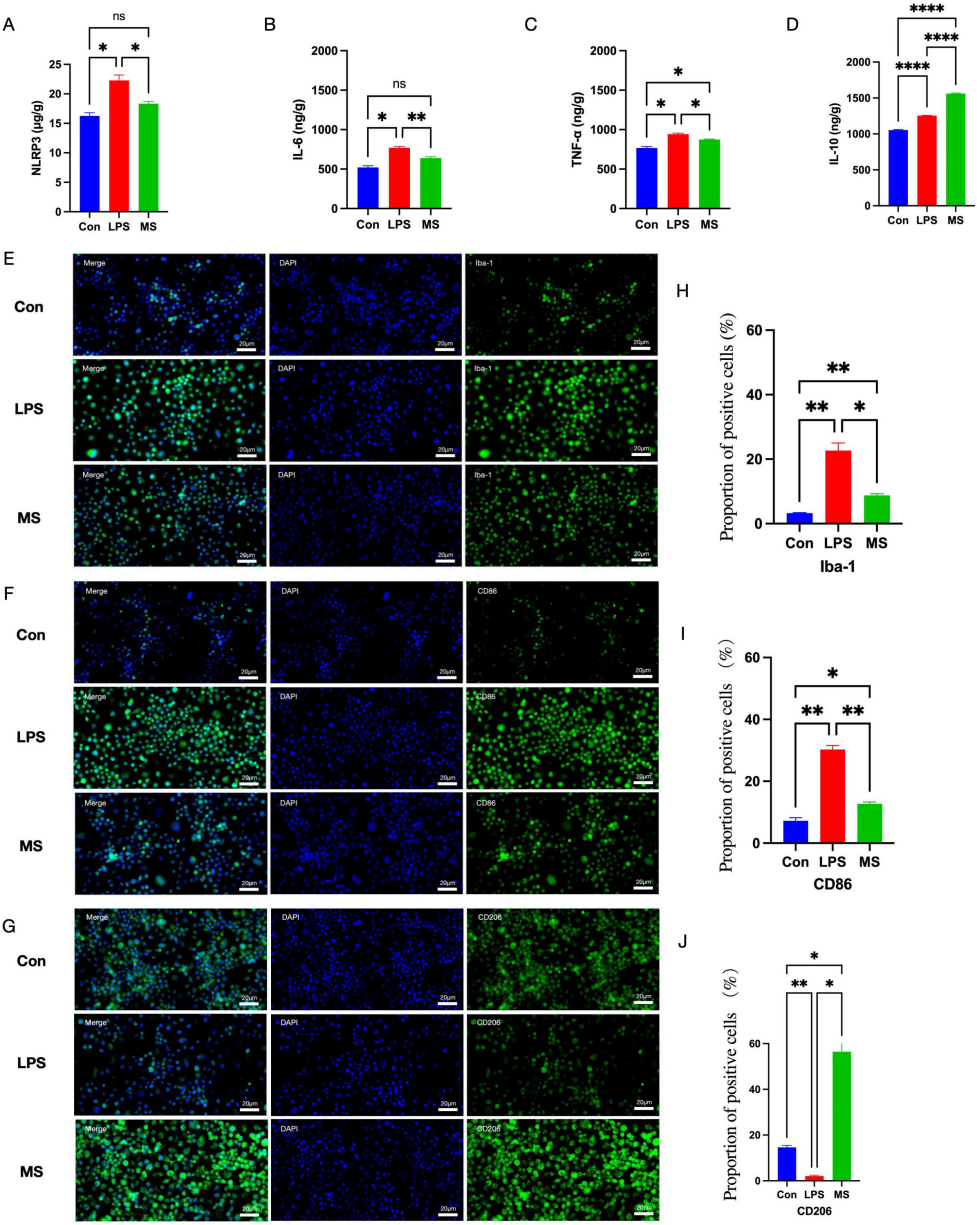
Effects of rTMS on neuroinflammation and the M1/M2 polarization state of microglia *in vitro*. **(A–D)** Elisa was used to examine the expression levels of NLRP3, IL-6, TNF-α and IL-10 expression level. **(E–G)** Immunofluorescence staining of Iba-1, CD86, CD206. **(H–J)** Proportion of positive cells for target proteins. The number of samples in each group was 3. The results was analyzed with one-way ANOVA followed by Tukey’s *post-hoc* test, *p < 0.05, **P < 0.01 and ****P < 0.0001.

*In vitro* experiments further corroborated the findings reported above. In comparison with the Con group, the LPS group showed elevated levels of NLRP3, IL-6, and TNF-α and decreased levels of IL-10 (p < 0.05) (**Figures 4A–D**), the microglial marker Iba-1 increased (p < 0.01), as well a significantly higher proportion of CD86-positive cells (p < 0.05) and a significantly lower proportion of CD206-positive cells (p < 0.01) (**Figures 4E–J**). After 2 days of magnetic stimulation, the levels of NLRP3, IL-6, and TNF-α in the MS group decreased (p < 0.05) and the IL-10 level increased (p < 0.01). Additionally, the MS group showed a marked reduction in Iba-1 and CD86 expression and a significant increase in CD206 expression (p < 0.05) (**Figures 4E–J**).

### 3.3 Magnetic stimulation downregulates METTL3, m6A methylation, NMDAR2B, and YTHDF1

**Figure 5:**
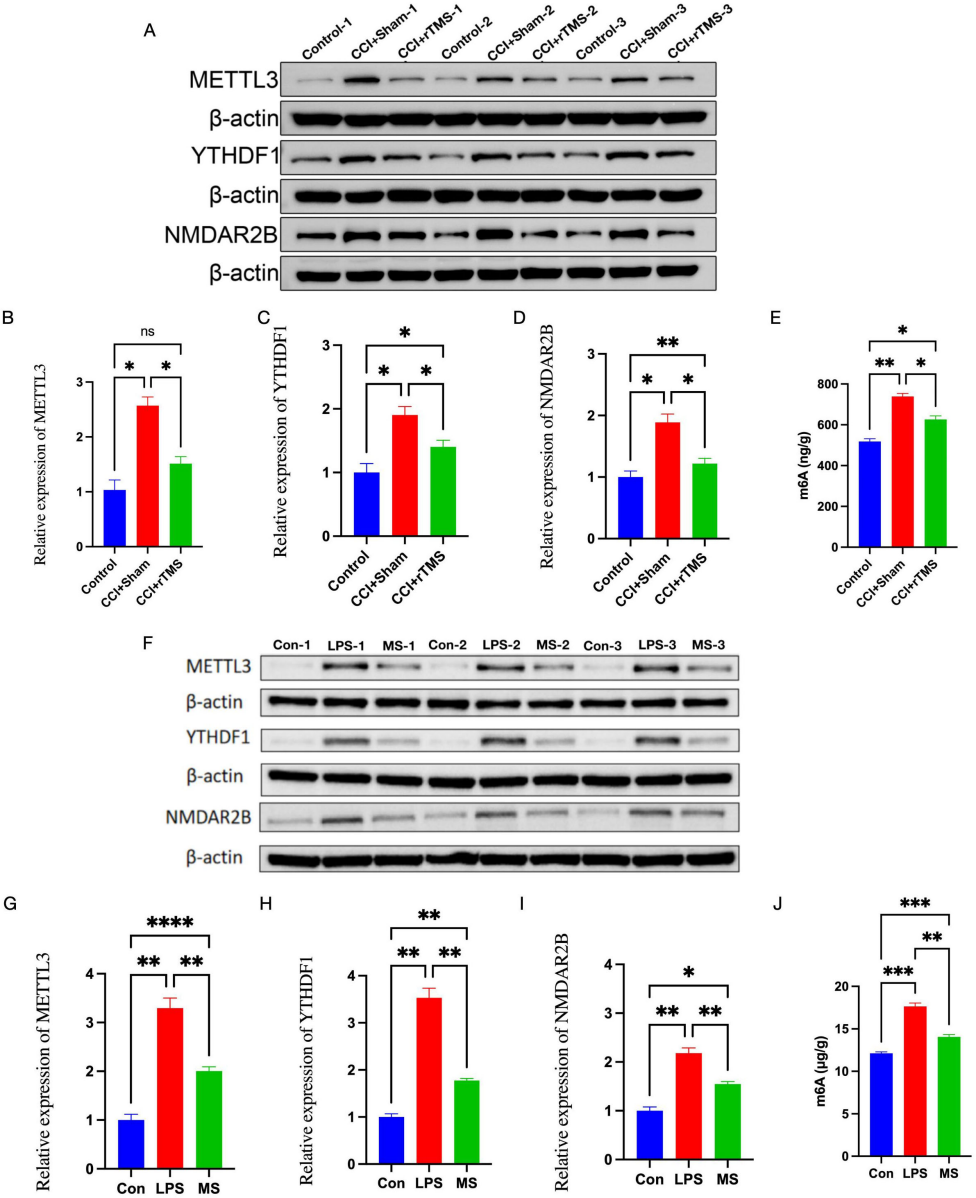
*In vivo***(A–E)** and *in vitro***(F–J)** effects of rTMS on METTL3, m6A methylation, NMDAR2B, and YTHDF1. **(A)** METTL3, NMDAR2B, YTHDF1 protein bands in the DLPFC. **(B–D)** Western blot analysis of METTL3, YTHDF1, and NMDAR2B protein expression in the DLPFC. **(E)** ELISA analysis of the methylation modification level of m6A in the DLPFC. The number of samples in each group is 3. **(F)** METTL3, NMDAR2B, YTHDF1 protein bands in BV2 cells. **(G–J)** Western blot analysis of METTL3, YTHDF1, and NMDAR2B protein expression levels in BV2 cells. (P) ELISA analysis of the methylation modification level of m6A in BV2 cells. The number of samples in each group was 3. The results was analyzed with one-way ANOVA followed by Tukey’s *post-hoc* test, *p < 0.05, **P < 0.01, ***P < 0.001 and ****P < 0.0001.

*In vivo*, western blot, and qRT-PCR analyses revealed that in comparison with the Control group, the CCI+Sham group exhibited significantly higher levels of METTL3, m6A methylation, NMDAR2B, and YTHDF1 in the DLPFC (p < 0.05). After 4 weeks of rTMS treatment, the CCI+rTMS group showed markedly reduced levels of these markers (p < 0.05) (**Figures 5A–E** and **Supplementary Figures S1A–C**).

*In vitro* experiments confirmed the effects of magnetic stimulation on LPS-induced microglial cells. In comparison with the Con group, the LPS group exhibited increased levels of METTL3, m6A, NMDAR2B, and YTHDF1 (p < 0.01). After 2 days of magnetic stimulation, the MS group showed decreased levels of these markers (p < 0.01), consistent with the *in vivo* observations (**Figures 5F–J**, **Supplementary Figures S1A–C**).

### 3.4 Roles of METTL3 and NMDAR2B in regulating microglial polarization by magnetic stimulation

**Figure 6:**
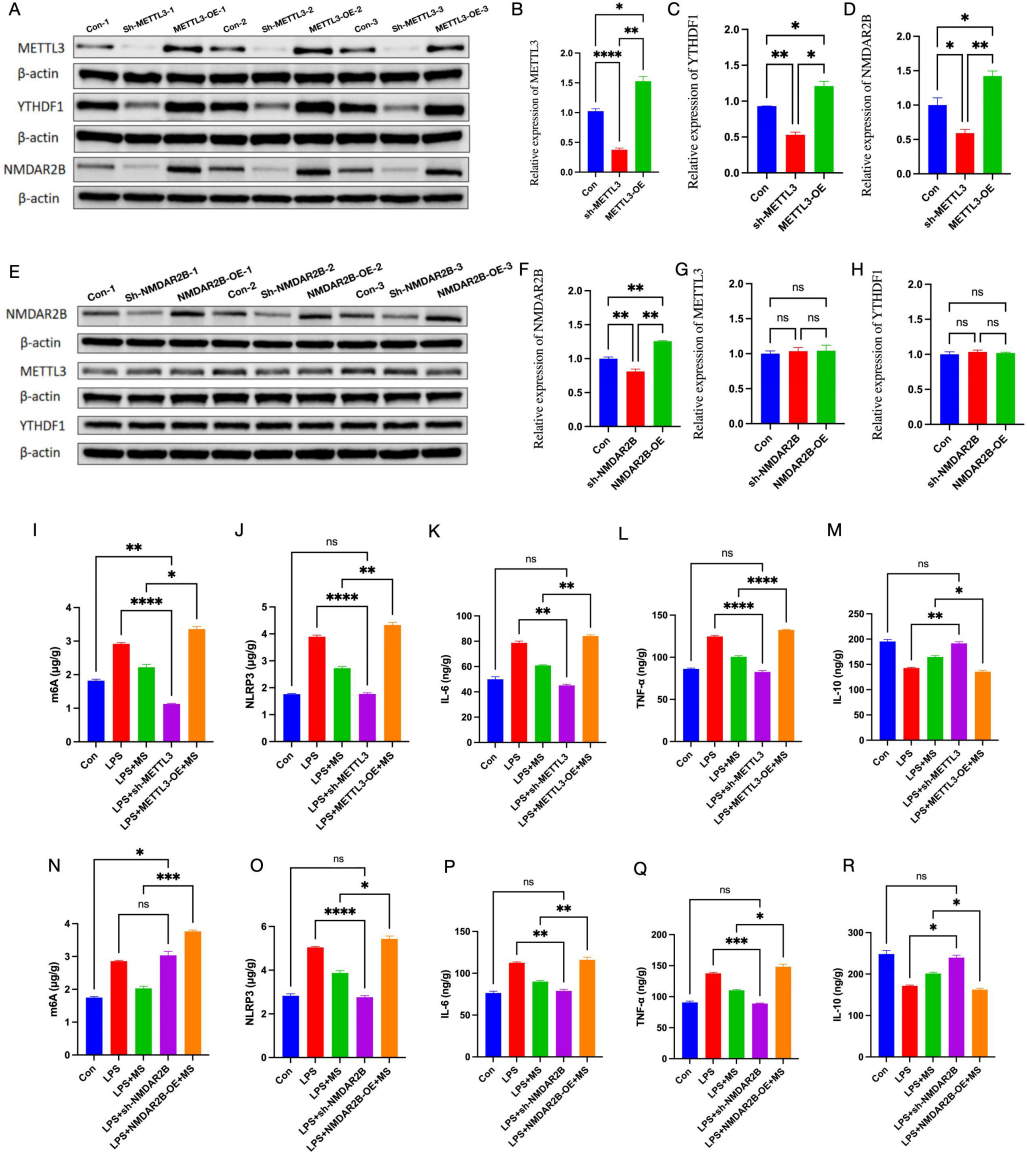
The roles of METTL3 and NMDAR2B in the anti-inflammatory effects of magnetic stimulation *in vitro*. **(A)** Effects of inhibition and overexpression of METTL3 on the protein bands of METTL3, NMDAR2B, and YTHDF1. **(B–D)** Western blot analyses of METTL3, YTHDF1, and NMDAR2B protein expression. **(E)** Effects of inhibition and overexpression of NMDAR2B on the protein bands of METTL3, NMDAR2B, and YTHDF1 **(F–H)** Western blot analyses of METTL3, YTHDF1, and NMDAR2B protein expression. **(I–R)** ELISA was used to detect the methylation modification level of m6A, NLRP3, IL-6, TNF-α, and IL-10. The number of samples in each group was 3. The results was analyzed with one-way ANOVA followed by Tukey’s *post-hoc* test, *p < 0.05, **P < 0.01, ***P < 0.001 and ****P < 0.0001.

To further elucidate the roles of METTL3 and NMDAR2B in magnetic stimulation-mediated microglial polarization and neuroinflammation, pharmacological inhibitors and agonists were used, and challenged with LPS to evoke neuroinflammation, and subsequently subjected to magnetic-stimulation intervention.in the vitro experiments. Compared with the Con group, METTL3 inhibition reduced YTHDF1 and NMDAR2B expression (p < 0.05). Overexpression of METTL3 elevated YTHDF1 and NMDAR2B levels (p < 0.05). Inhibition or overexpression of NMDAR2B did not alter METTL3 or YTHDF1 expression (**Figures 6A–H**).

**Figure 7:**
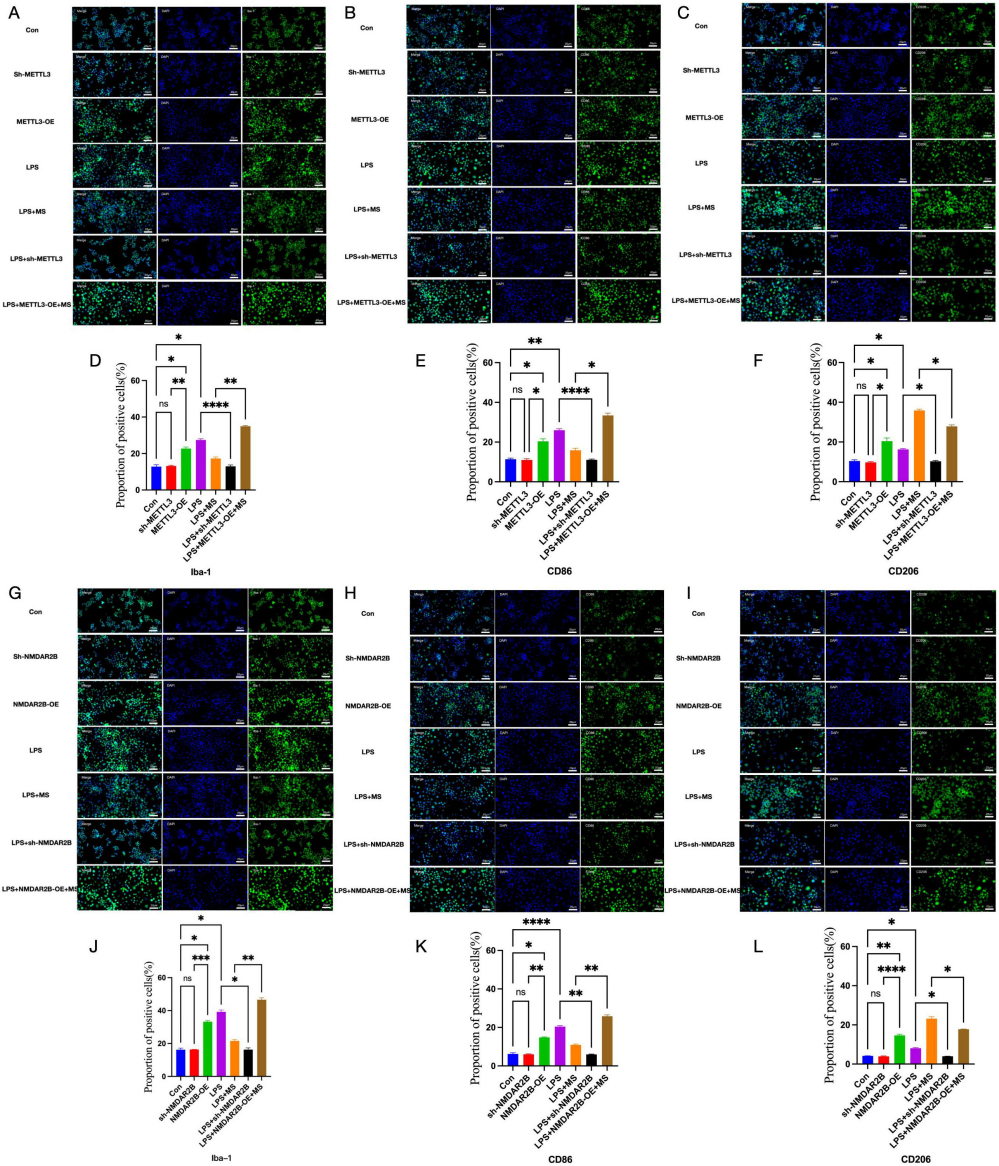
The roles of METTL3 and NMDAR2B in the regulation of microglial cell polarization by magnetic stimulation *in vitro*. **(A–C, G–I)** Immunofluorescence staining of Iba-1, CD86 and CD206. **(D–F, J–L)** Proportion of positive cells for target proteins. The number of samples in each group was 3. The results was analyzed with one-way ANOVA followed by Tukey’s *post-hoc* test, *p < 0.05, **P < 0.01, ***P < 0.001 and ****P < 0.0001.

Compared with the LPS group, the LPS+sh-METTL3 and LPS+sh-NMDAR2B groups showed the levels of m6A, NLRP3, IL-6, and TNF-α dncreased(p < 0.05), while the IL-10 level increased (p < 0.05). And reduced proportions of Iba-1, CD86 and CD206-positive cells(p < 0.05). Furthermore, Compared with the Con group, there was no statistical difference in Iba-1, CD86 and CD206 between the sh-METTL3 group and the Sh-NMDAR2B group, conversely, METTL3-OE and NMDAR2B-OE significantly elevated the expression of Iba-1, CD86 and CD206 (p < 0.05). Those indicating a key role of METTL3 and NMDAR2B in M1 polarization of microglial and the neuroinflammation (**Figures 6I–R**, **7**).

In contrast, the LPS+METTL3-OE+MS and LPS+NMDAR2B-OE+MS groups exhibited more Iba-1 and CD86-positive cells, lower CD206 expression, elevated levels of m6A, NLRP3, IL-6, and TNF-α (p < 0.05), and reduced IL-10 levels (p < 0.05) in comparison with the LPS+MS group. These results suggest that the M2-polarizing and anti-neuroinflammatory effects of magnetic stimulation were reversed by METTL3 and NMDAR2B overexpression (**Figure 7**).

## 4 Discussion

In this study, we deliberately adopted a “phenotype-to-molecule” design. Immediately after the 4-week rTMS regimen, comprehensive behavioral testing was conducted to confirm CCI-induced pain and depression and to validate the therapeutic efficacy of rTMS. These data—presented in the first Results section—establish the biological relevance that guided all subsequent molecular analyses. Consistent with our previous findings (8). The rTMS significantly improved pain-related outcomes, as evidenced by increases in PWMT, PWL, and SFI. Moreover, rTMS ameliorated depressive-like behaviors, reflected by reduced immobility time in the forced swim test and increased preference for novel objects.

At the molecular level, magnetic stimulation treatment reduced the expression of Iba-1 and CD86 while increasing the expression of CD206 in CCI rats and LPS-stimulated BV2 microglial cells. It also decreased the levels of IL-6 and TNF-α, elevated IL-10 levels, and downregulated m6A methylation as well as the expression of METTL3, YTHDF1, and NMDAR2B. To further clarify the roles of METTL3 and NMDAR2B, we conducted additional experiments involving targeted inhibition and activation of these molecules. The results confirmed that both METTL3 and NMDAR2B are key mediators of the effects of magnetic stimulation on microglial polarization and neuroinflammation.

As a representative non-invasive neuromodulation technique, the therapeutic efficacy of rTMS for NeuP has been increasingly supported by numerous recent clinical studies and secondary analyses, including meta-analyses (45, 46).

Regarding the choice of stimulation site, the effectiveness of stimulation at the primary motor cortex is supported by a substantial body of evidence. Among other regions, the DLPFC has been the most extensively studied. From a functional perspective, the DLPFC serves as a critical node within multiple brain networks, participating in cognitive, emotional, and sensory processing (47). Some studies have shown that patients with chronic pain usually exhibit more functional abnormalities in this area, and these abnormalities are thought to modulate pain perception, likely through the activation of brainstem descending pain regulatory pathways (48). Consistent with these findings, a sham-controlled study found that rTMS applied to the DLPFC significantly reduced pain intensity scores induced by laser stimulation (49). Furthermore, a clinical trial involving patients with NeuP following spinal cord injury demonstrated that rTMS targeting the DLPFC moderately alleviated pain, improved sleep quality, and enhanced emotional states (50). Therefore, considering the close interaction between pain and emotional processing, we selected the PFC region as our intervention target in this experiment.

We adopted the 4-week, 10 Hz (8) left-DLPFC protocol because it is the best-supported regimen for neuropathic pain (NeuP) relief. A meta-analysis of 14 randomized trials (n = 574) showed that high-frequency rTMS over DLPFC yields a large pain-reduction effect (SMD = –1.02, 95% CI –1.37 to –0.67) with durable benefits up to 4 weeks post-treatment (51). Clinically, our group previously applied the identical schedule (10 Hz, 120% RMT, 5 days for 4 weeks) to 48 NeuP patients and observed a 46% decrease in VAS scores versus 12% under sham (p < 0.01) (52). Mechanistically, 10 Hz DLPFC stimulation up-regulates anterior-cingulate metabolism and increases serum BDNF and NGF levels, reversing maladaptive plasticity that underlies chronic pain. These converging clinical and basic findings justify the present stimulation paradigm (53). Numerous clinical studies have confirmed the therapeutic efficacy of 10 Hz rTMS for NeuP. Hodaj et al. administered 10 Hz rTMS over three weeks to 57 patients with chronic NeuP, and some patients achieved a pain reduction of ≥30% on the visual analog scale (VAS), which was accompanied by a notable increase in intracortical facilitation (ICF) (110.3% ± 12.5% vs. 170.0% ± 28.3%, p = 0.039) (54). Yilmaz et al. reported that 10 Hz rTMS treatment delivered over 10 days significantly alleviated NeuP following spinal cord injury (VAS score: 1.0 ± 1.0 vs. 3.0 ± 2.0, p > 0.05) (55). Our stimulation parameters (10 Hz, 100% resting motor threshold, 1000 pulses/session, 5 days/week for 4 weeks, left dorsolateral prefrontal cortex) are consistent with the FDA-approved HF-rTMS protocol for treatment-resistant depression and align with recent neuropathic pain trials that achieved ≥30% pain reduction (56, 57). Next, we consider whether METTL3 or NMDAR2B could serve as response biomarkers. Finally, the selective METTL3 catalytic inhibitor STM2457 is currently in Phase I oncology safety trials (58). Preclinical studies show that intranasal STM2457 penetrates the rodent central nervous system and reduces brain m6A levels by 26% within 4 hours. Combining low-dose STM2457 with fewer rTMS sessions could offer a session-sparing strategy, extending microglial M2 polarization while limiting clinical visits—an hypothesis worthy of dedicated investigation (59).

Neuroinflammation, which is mediated by microglial polarization, plays a crucial role in the onset and progression of NeuP. Therefore, it may represent a key mechanism through which magnetic stimulation alleviates pain. In the *in vivo* experiments, magnetic stimulation significantly downregulated CD86 while upregulating CD206, thereby reducing M1 polarization and increasing M2 polarization in microglia and improving neuroinflammation. These findings are consistent with the findings of our previous study (22). Furthermore, the present study confirmed these effects *in vitro* using LPS-induced BV2 microglia, reinforcing the role of microglial polarization in the therapeutic effects of magnetic stimulation (60). Indeed, accumulating evidence suggests that regulating microglial polarization may be a central mechanism by which rTMS exerts its therapeutic effects across various diseases. Zuo et al. demonstrated that rTMS suppressed microglial activation in a mouse model of depression, reduced the release of pro-inflammatory cytokines, and alleviated neuroinflammation (61). Hong et al. found that high-frequency rTMS inhibited M1 microglial polarization in a mouse model of ischemic stroke (middle cerebral artery occlusion [MCAO]), thereby reducing neuroinflammation (43). Additionally, Wang et al. reported that rTMS treatment for one week in mice with middle cerebral artery occlusion/reperfusion (MCAO/R) injury improved balance and motor coordination. Concurrently, rTMS downregulated pro-inflammatory M1 microglial activation (Iba1+/CD86+) and upregulated anti-inflammatory M2 activation (Iba1+/CD206+) in the peri-infarct region, thereby significantly altering the M1/M2 phenotype ratio (62). Collectively, these findings suggest that regulating neuroinflammation through microglial polarization may represent a crucial pathway through which rTMS exerts its therapeutic effects, including its anti-NeuP actions.

Although we focused on microglia, the METTL3/m^6^A/NMDAR2B/NLRP3 axis also operates across cell types: neurons supply NMDAR2B that can be m^6^A-modified and shape microglial activation (63), high-frequency rTMS boosts BDNF, PSD-95 and synapsin I while restoring glutamate/GABA balance (64), and GABAergic activation increases neuronal Cx3cl1 to indirectly modulate microglia (65). Astrocytes are equally engaged: after TBI, rTMS down-regulates IL-6/TNF-α, up-regulates IL-10, and modulates P75NTR/IL-33 in astrocytes, while Ca²^+^-dependent gliotransmitter release supports synaptic repair (66). These converging neuron–glia networks underscore that rTMS analgesia is a multicellular process extending beyond microglia, thereby clarifying the cell-autonomous gatekeeper mechanisms that initiate the multicellular analgesic cascade.

We observed significant upregulation of METTL3, m6A methylation, YTHDF1, and NMDAR2B in both the CCI rat model and LPS-stimulated microglia. After rTMS intervention, the pain and depression-related behaviors of SD rats improved, and the levels of METTL3, m6A methylation, YTHDF1, and NMDAR2B were all lower than those of CCI rats. Subsequent *in vitro* experiments revealed that inhibiting METTL3 or NMDAR2B effectively reversed LPS-induced neuroinflammation and M1 microglial polarization. In contrast, overexpression or activation of METTL3 or NMDAR2B counteracted the anti-inflammatory effects of magnetic stimulation, impairing its ability to promote M2 polarization. These findings suggest that METTL3 and NMDAR2B may serve as key molecular targets through which rTMS modulates microglial polarization and alleviates neuroinflammation.

Accumulating evidence indicates that methylation modifications play a critical role in the development and maintenance of NeuP. For instance, Su et al. demonstrated that METTL3 promotes LPS-induced microglial M1 polarization and subsequent neuroinflammation (67). Wu et al. reported that in rats with traumatic brain injury, the expression of METTL3 was elevated, microglia were activated, and M1 polarization increased (68). Cheng et al. further confirmed that METTL3 is involved in regulating microglial M1/M2 polarization (29). Beyond METTL3, the m6A axis is dynamically opposed by the demethylases ALKBH5 and FTO. Recent work has demonstrated that ALKBH5-driven m6A erasure promotes M1-to-M2 transition in microglia (69) and that FTO knock-down exacerbates LPS-induced neuroinflammation (70). Whether rTMS modulates these erasers in parallel to METTL3 is unknown. Our data show that 10 Hz stimulation reduces METTL3 expression and global m6A abundance (**Figure 5**), consistent with a Ca²^+^- and CREB-dependent mechanism (71). Indeed, rapid Ca²^+^ influx through rTMS-activated voltage-gated channels can de-phosphorylate CREB (72), thereby decreasing CREB occupancy on the METTL3 promoter. Mechanistically, we speculate—rather than conclude—that rTMS may down-regulate METTL3 through Ca²^+^-dependent signaling (73, 74). High-frequency (10 Hz) stimulation is known to open L-type Ca²^+^ channels (Cav1.2/1.3) within seconds, eliciting a cytosolic Ca²^+^ surge that can recruit calcineurin and reduce CREB-Ser133 phosphorylation (75). Whether this Ca²^+^/CREB axis is the principal route by which rTMS represses METTL3 transcription, or whether the field directly modulates METTL3 mRNA stability, alters its enhancer activity, or engages yet-unidentified pathways, remains experimentally unresolved. Parallel changes in demethylases (ALKBH5, FTO) could further shape the microglial m6A landscape. Combinatorial CRISPR-interference screens targeting CREB, ALKBH5 and FTO will be required to disentangle these possibilities (**Figure 5**).

In addition to METTL3, NMDAR2B has also been implicated in pain-related neuroinflammation. Guo et al. found that tyrosine phosphorylation of NMDAR2B in the spinal cord is associated with the development and maintenance of inflammatory hyperalgesia (76). Tang et al. demonstrated that overexpression of NMDAR2B in the forebrain exacerbates inflammation-related persistent pain (77). Supporting these findings, Kim et al. reported that the administration of NMDAR2B antagonists (e.g., ifenprodil and Ro25-6981) significantly alleviated NeuP in a mouse model of spinal cord injury (36). Moreover, a previous study demonstrated that NMDAR activation promotes M1 microglial polarization and sustains neuroinflammation (78), consistent with our current findings.

Collectively, these studies, together with our findings, suggest that both METTL3 and NMDAR2B play important roles in microglial polarization-mediated NeuP and may represent promising therapeutic targets for rTMS-based interventions.

Simultaneously, Ma et al. reported that deep brain stimulation in mice exposed to foot shock alleviated post-traumatic stress disorder-like behaviors and reduced METTL3 expression (38). Similarly, Zhang et al. found that rTMS treatment in a vascular dementia rat model improved learning and memory while decreasing NMDAR2B levels (79). In another study, Ba et al. demonstrated that rTMS ameliorated levodopa-induced dyskinesia in Parkinson disease (PD) and downregulated NMDAR2B expression (80). Additionally, Hu et al. observed that rTMS intervention in CCI rats improved pain and depression-like behaviors, which were accompanied by a reduction in NMDAR2B levels (8). These findings align with the results of our study, which showed that magnetic stimulation reduced the elevated expression of both METTL3 and NMDAR2B under neuroinflammatory conditions both *in vivo* and *in vitro*. These studies suggest that magnetic stimulation may affect the polarization of microglia by regulating the expression of METTL3 and NMDAR2B, thereby exerting its anti-NeuP effect.

Furthermore, we found that NMDAR2B expression is regulated by METTL3, indicating that NMDAR2B may function as a downstream target of METTL3. This interaction plays a role in regulating microglial polarization and the pathogenesis of NeuP. Supporting this, Wang et al. demonstrated that in a rat model of early-stage Alzheimer’s disease, METTL3-mediated m6A modification enhances the stability of circRIMS2, which in turn regulates GluN2B (i.e., NMDAR2B) to cause synaptic dysfunction and memory impairment (81). These findings are consistent with our results and further support the involvement of the METTL3–NMDAR2B axis in microglial-mediated neuroinflammation and chronic pain.

Kui et al. found that the NLRP3 inflammasome antagonist MCC950 can effectively alleviate pain hypersensitivity in CCI rats (82). Similarly, Song et al. observed that NMDAR2B may promote depression-like behaviors by regulating NLRP3 expression in an LPS-induced mouse model of depression (35). Consistent with these findings, our experimental results demonstrated that inhibiting METTL3 or NMDAR2B led to decreased NLRP3 expression; on the other hand, activation of these molecules resulted in increased NLRP3 levels. Therefore, we speculate that NLRP3 may function as a downstream regulatory molecule of the METTL3/NMDAR2B pathway, participating in microglial polarization and neuroinflammation.

In our study, magnetic stimulation intervention significantly reduced NLRP3 expression, decreased the levels of the pro-inflammatory cytokines IL-6 and TNF-α, increased anti-inflammatory cytokine IL-10, and enhanced M2 microglial polarization. We hypothesize that NLRP3 acts as a downstream effector of METTL3/NMDAR2B, mediating the regulatory effects of magnetic stimulation on microglial polarization, thereby ameliorating neuroinflammation and alleviating NeuP.

Supporting this hypothesis, Wang et al. demonstrated that NLRP3 inhibition reduced microglial M1 polarization while increasing M2 polarization (83). Huang et al. reported that rTMS improved swallowing function in mice with PD by inhibiting NLRP3 (84). Zhang et al. found that activation of the NLRP3 inflammatory pathway may be an intrinsic factor in NeuP development and that rTMS treatment in spinal nerve ligation mice suppressed NLRP3 expression, thereby alleviating NeuP (85). These findings are consistent with our results and further support the role of NLRP3 in microglial-mediated neuroinflammation.

## 5 Conclusions

This study investigated the mechanism by which rTMS modulates microglial polarization by regulating METTL3-mediated m6A methylation. Our findings revealed that the METTL3/NMDAR2B/NLRP3 signaling axis plays a key role in the pathogenesis and progression of NeuP. Specifically, rTMS may alleviate NeuP and associated depression-like behaviors, at least in part, by downregulating METTL3 expression and m6A methylation levels. This, in turn, reduces YTHDF1-mediated translation of NMDAR2B, suppresses NLRP3 inflammasome activation, and promotes M2 polarization of microglia, ultimately modulating neuroinflammation.

## 6 Limitations

This study has some limitations. Firstly, we did not down-grade or overexpress METTL3 or NMDAR2B at the animal level to conduct *in vivo* validation of the METTL3/NMDAR2B axis. In the discussion section, we combined previous studies, such as gene knockout or pharmacological inhibition of METTL3 (such as STM2457) (86, 87) or NMDAR2B (88), and concluded that METTL3/NMDAR2B plays a significant role in rTMS regulating microglial M1/M2 polarization and neuroinflammation. Secondly, our research is limited to the DLPFC. However, brain function is a complex network regulation. It is still unknown whether the rTMS in the DLPFC area has an impact on other brain regions. Thirdly, translating our findings to patients is constrained by inter-species differences in skull thickness, coil-to-cortex distance, and the lack of validated METTL3/m6A biomarkers; larger, imaging-guided and sham-controlled clinical trials are essential to establish real-world efficacy.

## Data Availability

The original contributions presented in the study are included in the article/**Supplementary Material**. Further inquiries can be directed to the corresponding authors.

## Supplementary material

Supplementary Figure 1 **(A–C)** RT-PCR was used to detect the level of *METTL3*, *YTHDF1* and *NMDAR2B in vivo*, The number of samples in each group was 3. **(D–F)** RT-PCR was used to detect the level of *METTL3*, *YTHDF1* and *NMDAR2B in vitro* (n=3). The results was analyzed with one-way ANOVA followed by Tukey’s *post-hoc* test, *p < 0.05, **P < 0.01, ***P < 0.001 and ****P < 0.0001.

Supplementary Figure 2 Mechanism schematic diagram.
