# Supplementary figures and images for: Repetitive transcranial magnetic stimulation alleviates neuropathic pain via microglial polarization by modulating the METTL3/NMDAR2B/NLRP3 pathway
Source: Front Immunol. 2026 Jan 26;16:1666920. doi: 10.3389/fimmu.2025.1666920 (PMC12883363; doi:10.3389/fimmu.2025.1666920)

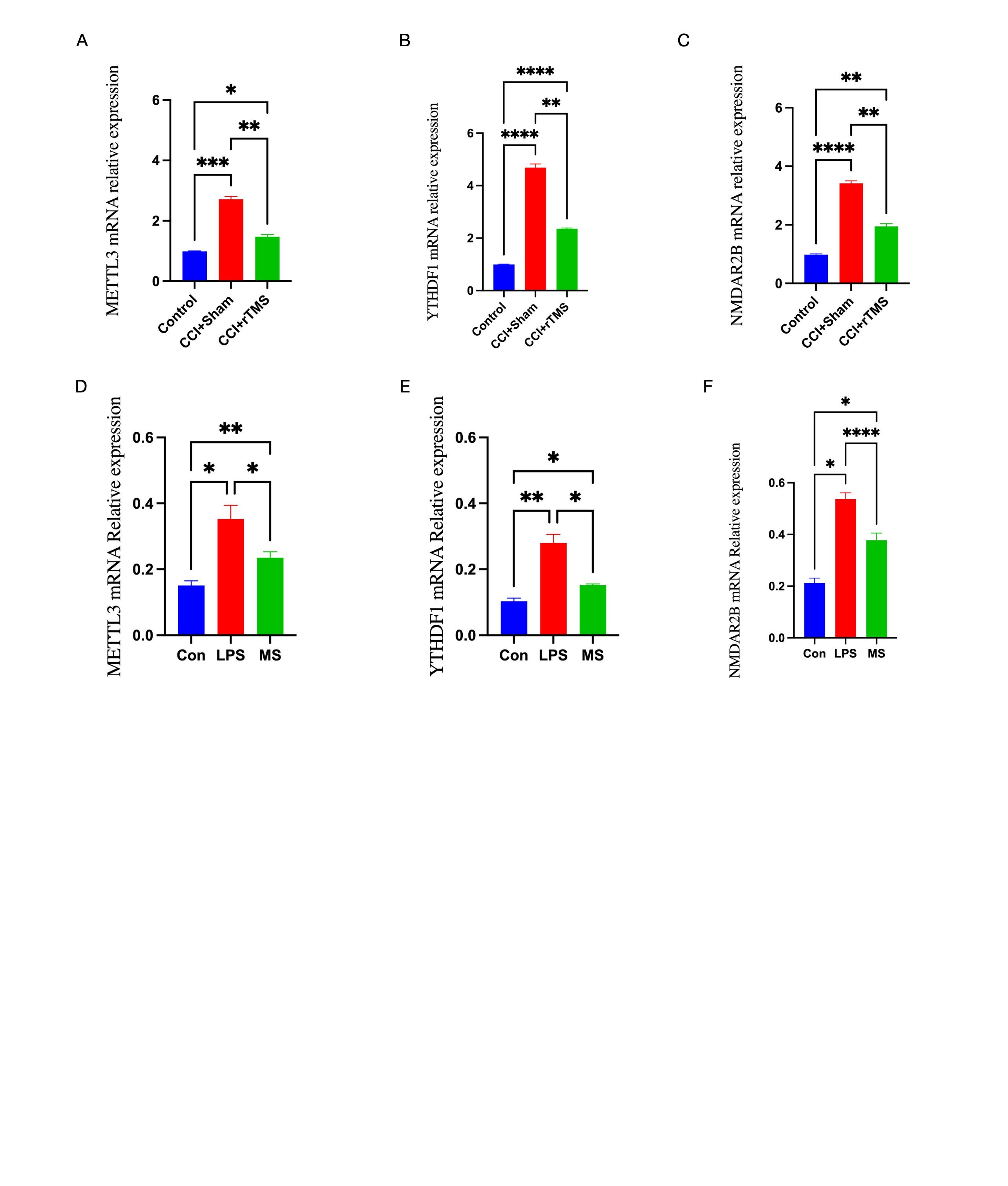

Supplement: Supplementary Figure 1 — (A–C) RT-PCR was used to detect the level of METTL3, YTHDF1 and NMDAR2B in vivo, The number of samples in each group was 3. (D–F) RT-PCR was used to detect the level of METTL3, YTHDF1 and NMDAR2B in vitro (n=3). The results was analyzed with one-way ANOVA followed by Tukey’s post-hoc test, *p < 0.05, **P < 0.01, ***P < 0.001 and ****P < 0.0001. [file Image1.jpeg]

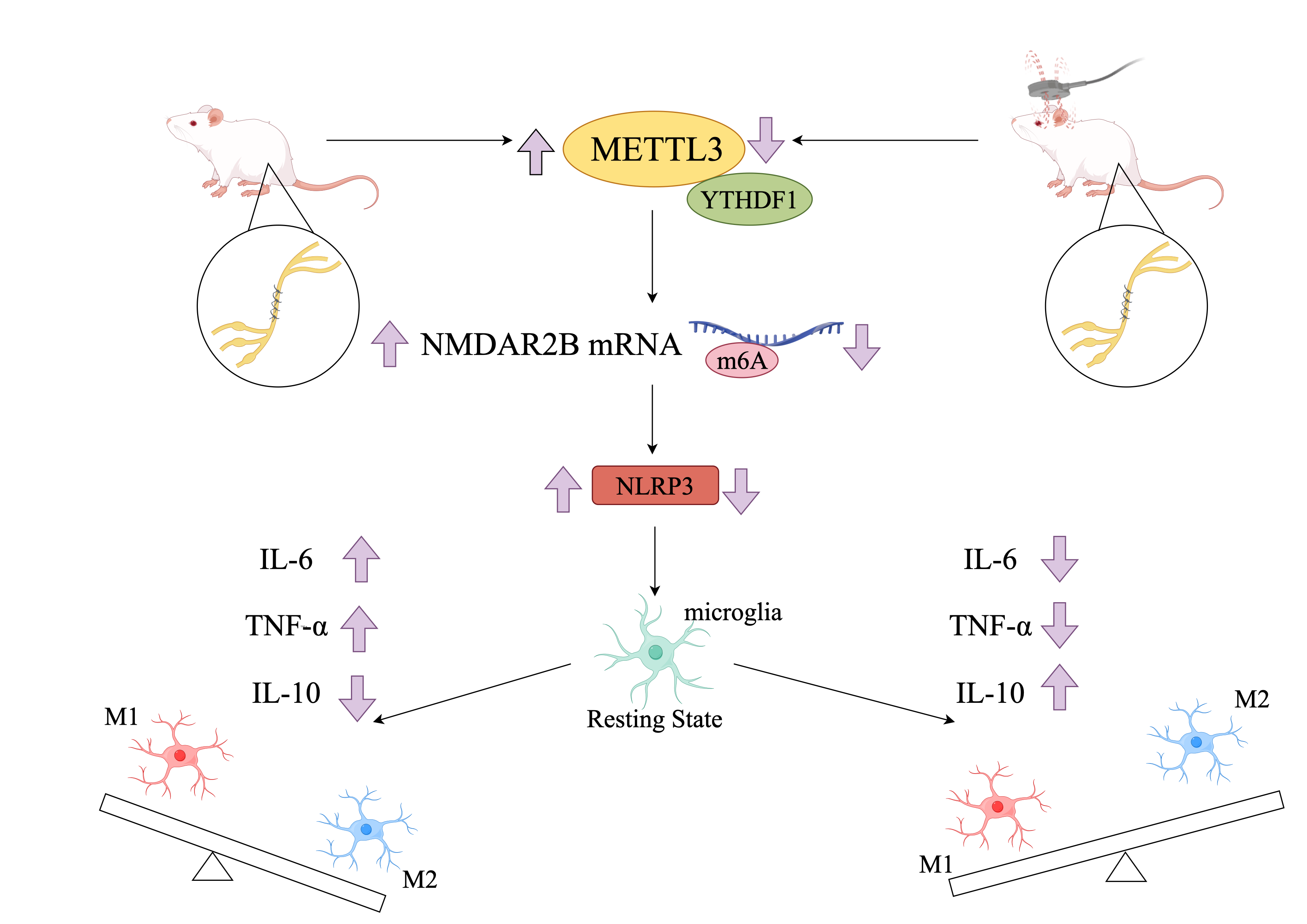

Supplement: Supplementary Figure 2 — Mechanism schematic diagram. [file Image2.png]

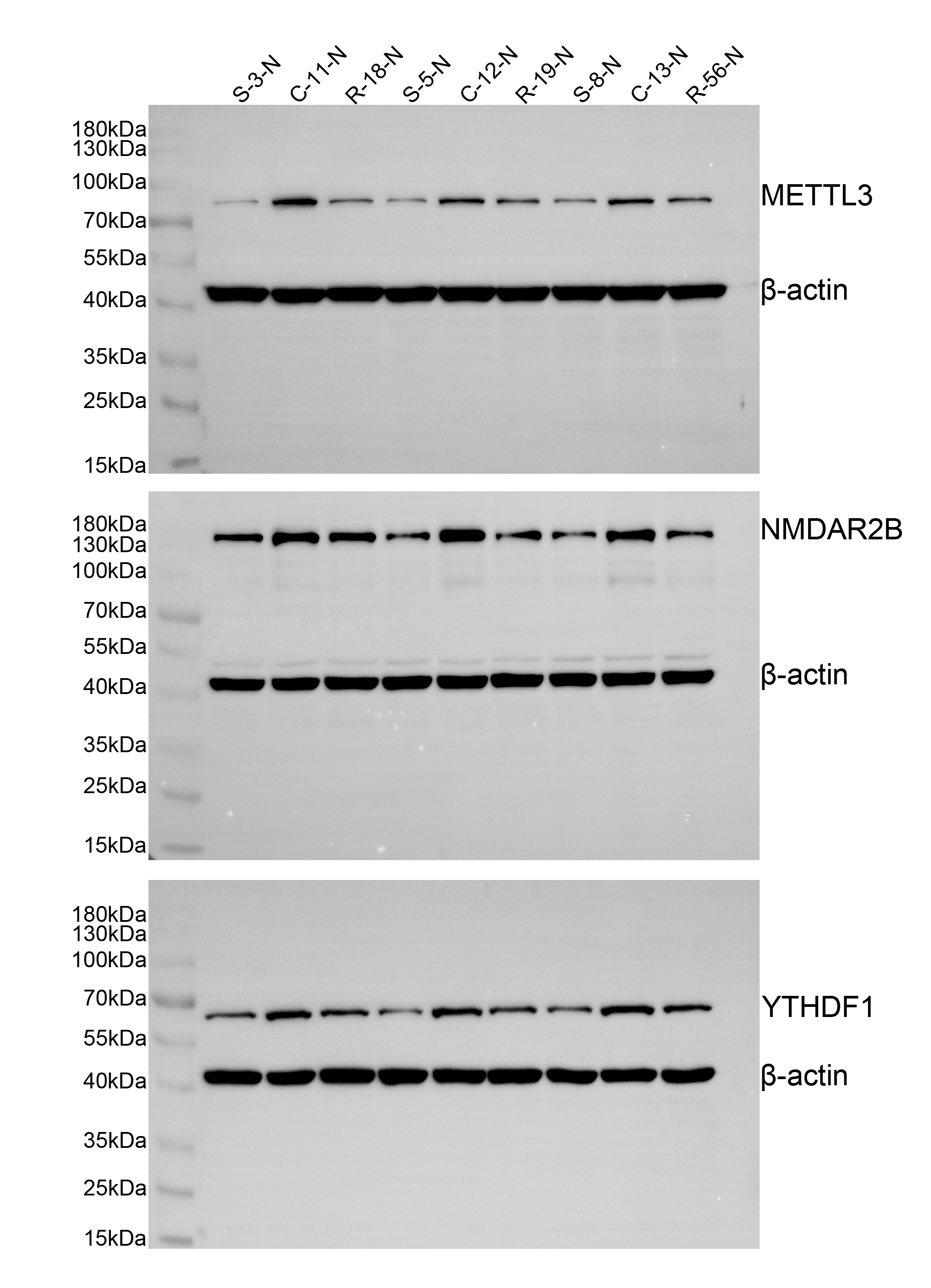

Supplement: Supplementary file 3 [file DataSheet1.zip › Original Images /Rat/METTL3NMDAR2BYTHDF1.tif]

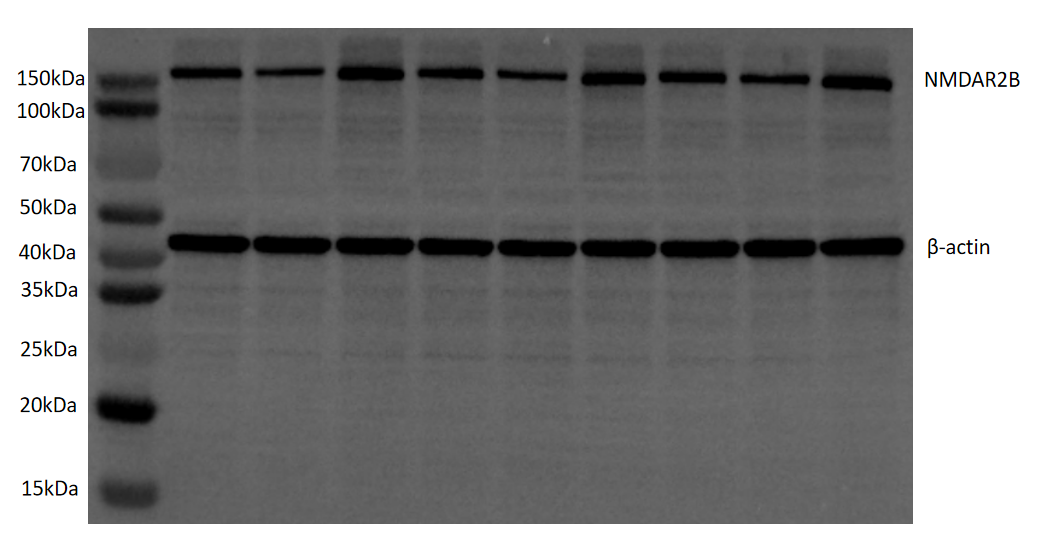

Supplement: Supplementary file 3 [file DataSheet1.zip › Original Images /BV2 Cells/Sh:OE-NMDAR2B/NMDAR2B.png]

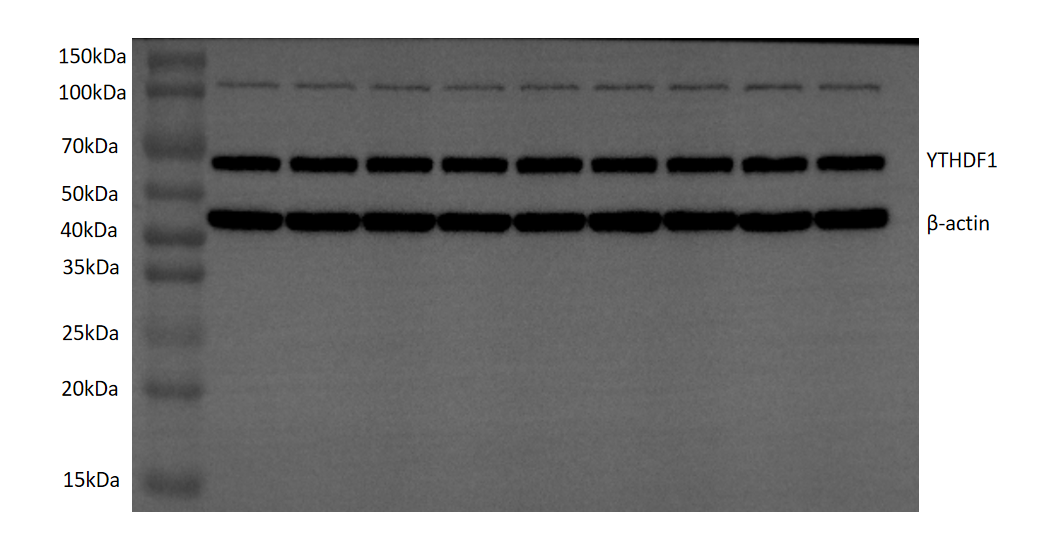

Supplement: Supplementary file 3 [file DataSheet1.zip › Original Images /BV2 Cells/Sh:OE-NMDAR2B/YTHDF1.png]

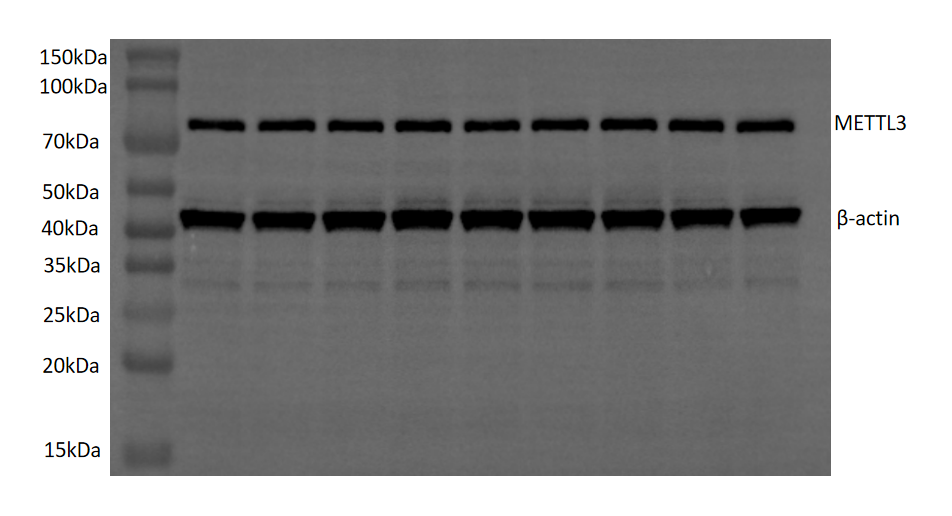

Supplement: Supplementary file 3 [file DataSheet1.zip › Original Images /BV2 Cells/Sh:OE-NMDAR2B/METTL3.png]

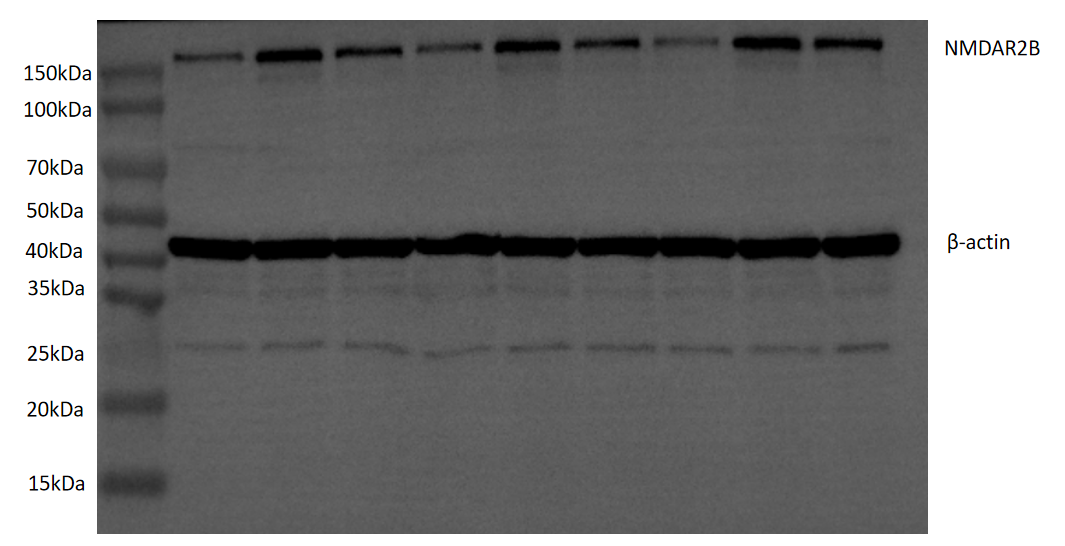

Supplement: Supplementary file 3 [file DataSheet1.zip › Original Images /BV2 Cells/1Con2LPS3MS/NMDAR2B.png]

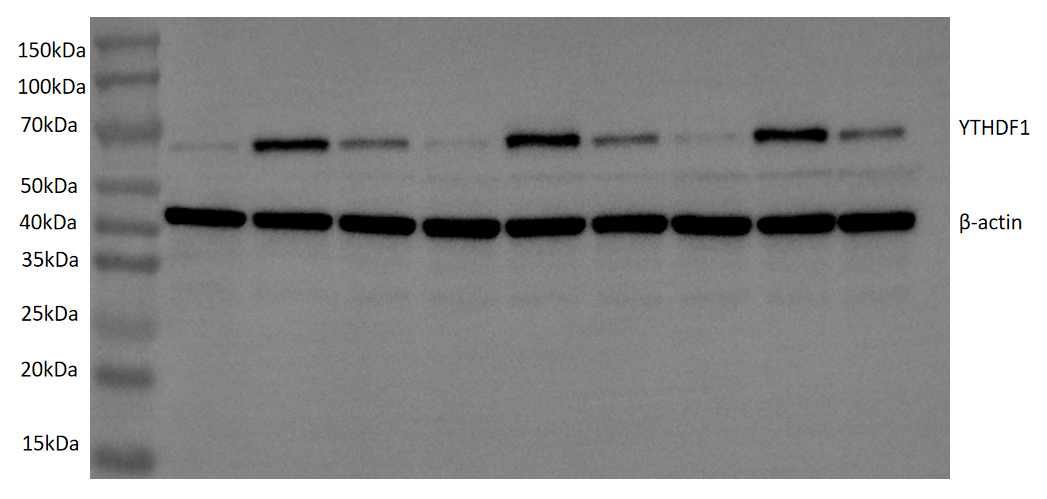

Supplement: Supplementary file 3 [file DataSheet1.zip › Original Images /BV2 Cells/1Con2LPS3MS/YTHDF1.png]

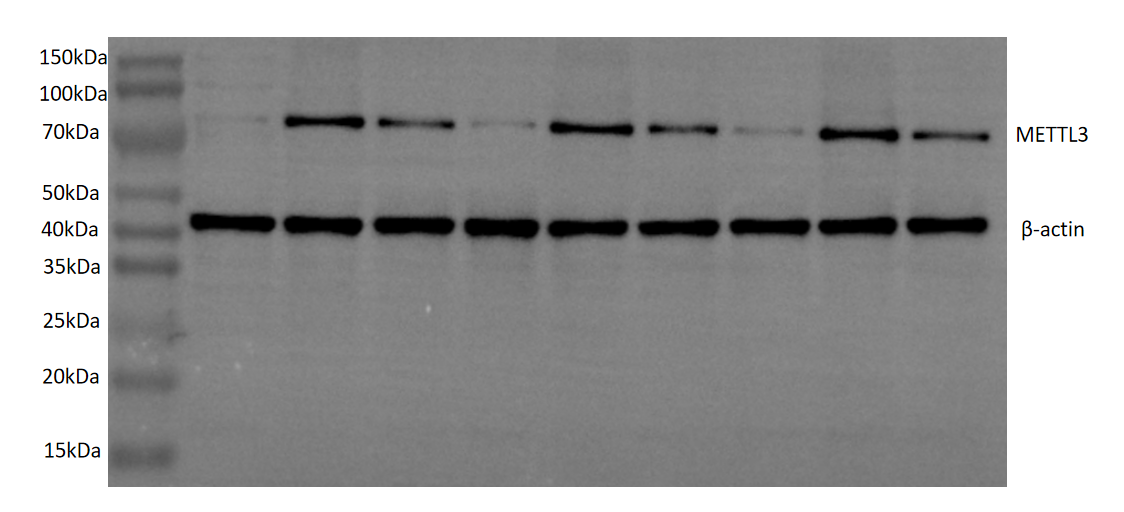

Supplement: Supplementary file 3 [file DataSheet1.zip › Original Images /BV2 Cells/1Con2LPS3MS/METTL3.png]

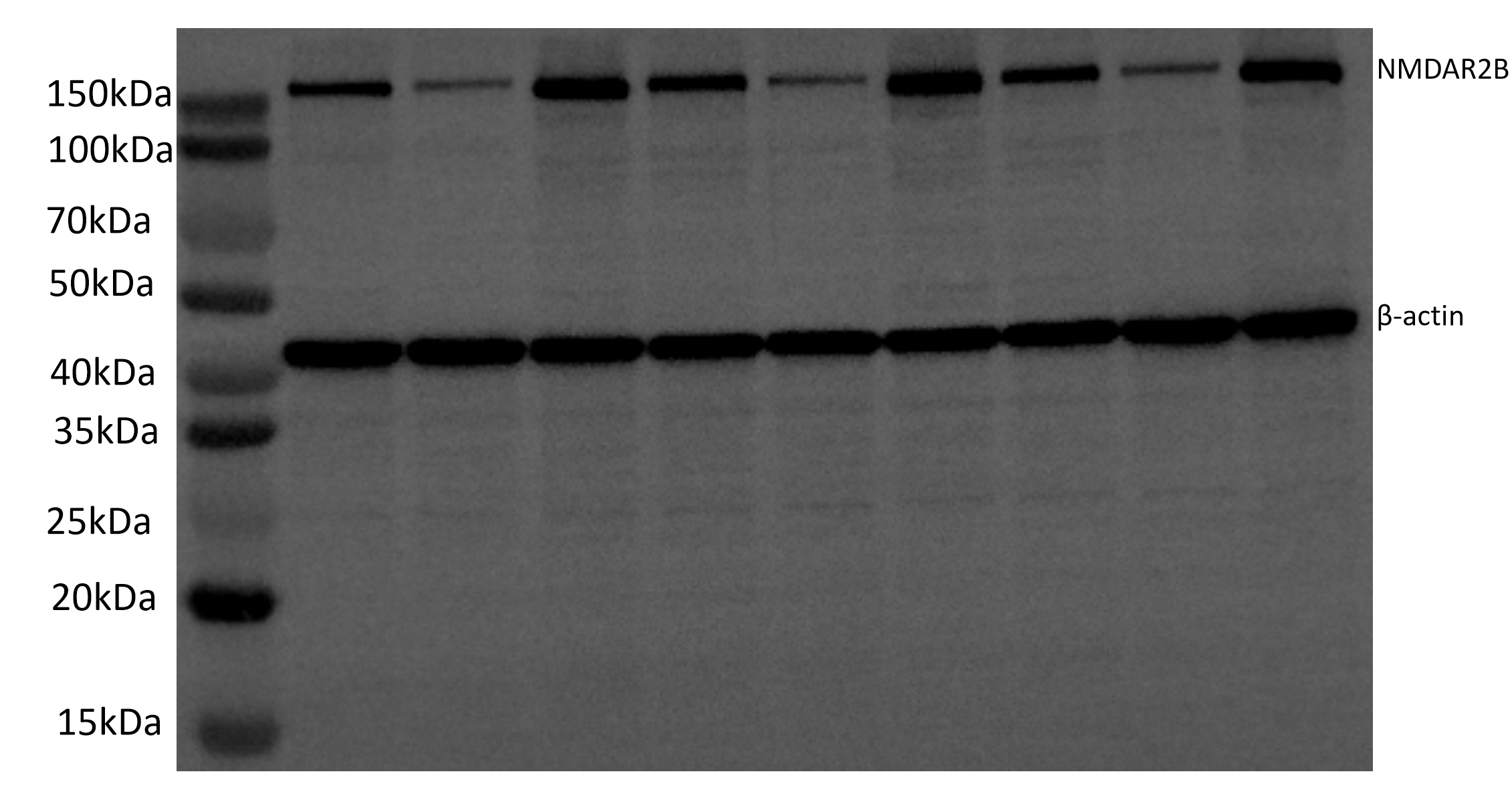

Supplement: Supplementary file 3 [file DataSheet1.zip › Original Images /BV2 Cells/Sh:OE-METTL3/NMDAR2B.png]

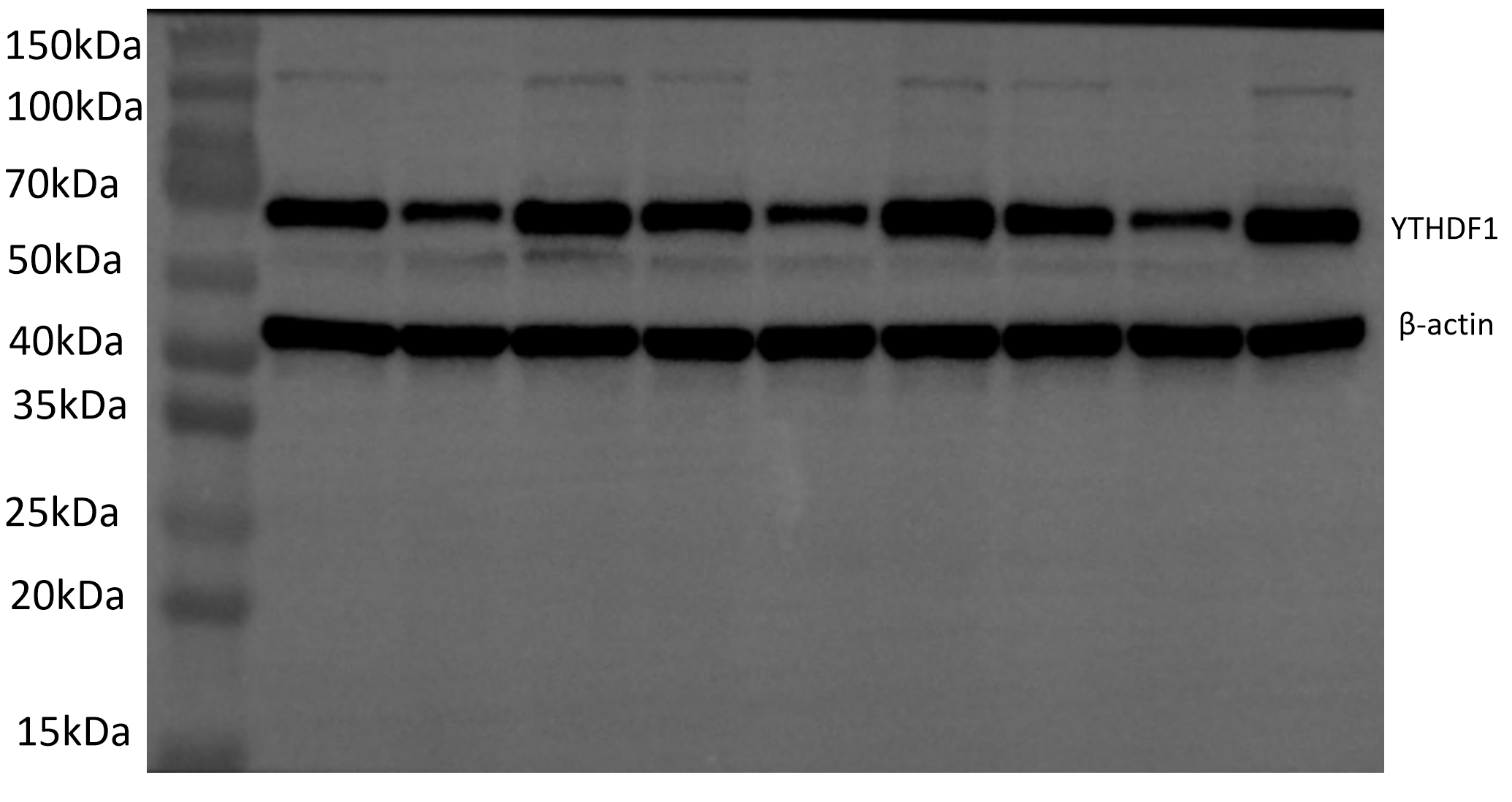

Supplement: Supplementary file 3 [file DataSheet1.zip › Original Images /BV2 Cells/Sh:OE-METTL3/YTHDF1.png]

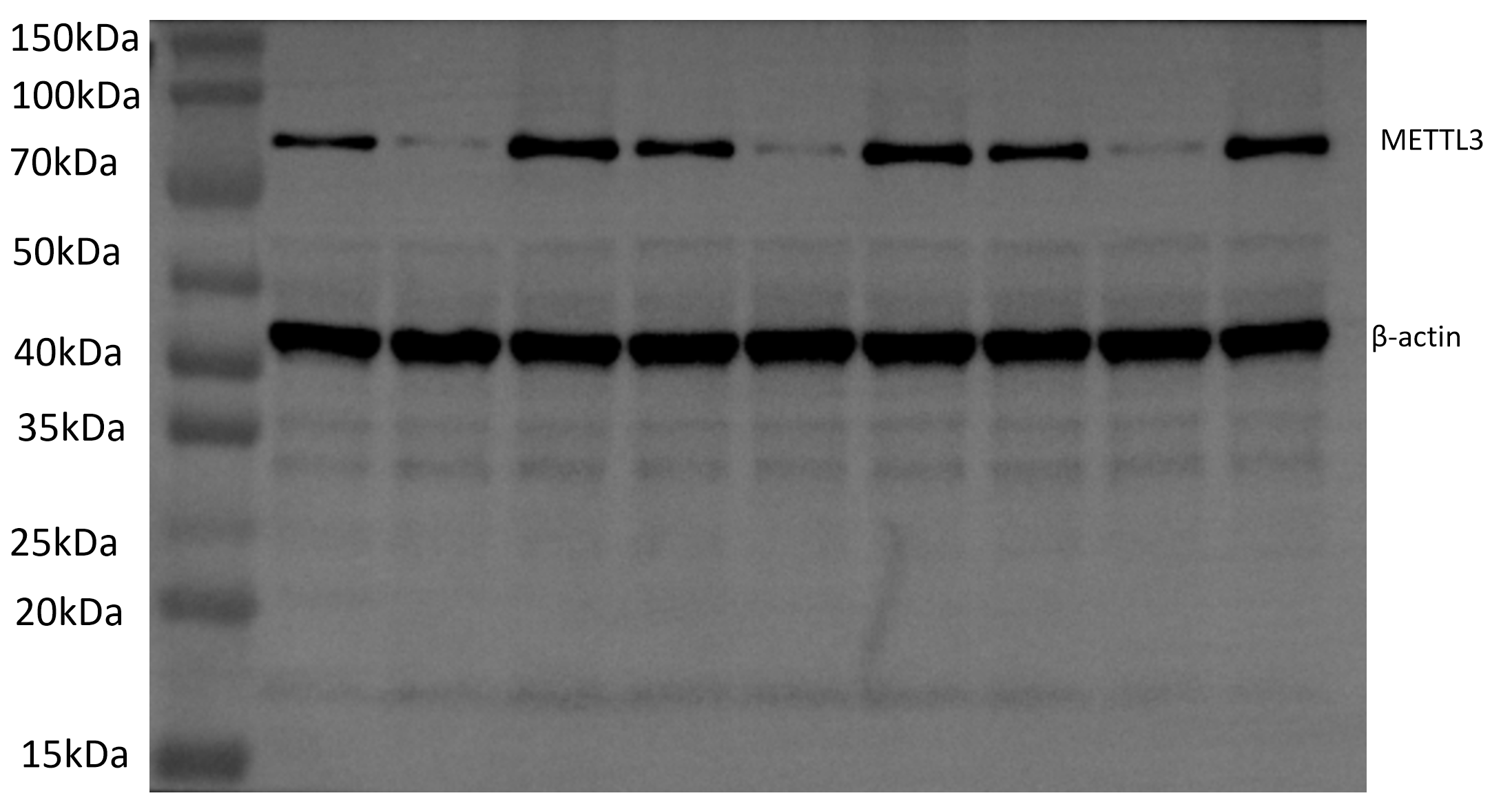

Supplement: Supplementary file 3 [file DataSheet1.zip › Original Images /BV2 Cells/Sh:OE-METTL3/METTL3.png]
